# Review of *Stantonia* Ashmead (Hymenoptera, Braconidae, Orgilinae) from Vietnam, China, Japan, and Russia, with descriptions of six new species

**DOI:** 10.3897/zookeys.723.21668

**Published:** 2017-12-18

**Authors:** Cornelis van Achterberg, Khuat Dang Long, Xue-xin Chen

**Affiliations:** 1 Department of Terrestrial Zoology, Naturalis Biodiversity Center, Postbus 9517, 2300 RA Leiden, The Netherlands; 2 Institute of Ecology & Biological Resources, Vietnam Academy of Science & Technology, 18 Hoang Quoc Viet Road, Cau Giay, Ha Noi, Vietnam; 3 Key Laboratory of Resource Biology and Biotechnology in Western China (Northwest University), Ministry of Education; School of Life Sciences, Northwest University, 229 North Taibai Road, Xi’an, Shaanxi 710069, China; 4 Institute of Insect Sciences, Zhejiang University, Zijingang Campus, Yuhangtang Road 866, Hangzhou 310058, China

**Keywords:** Braconidae, China, Japan, key, new species, Orgilinae, Russia, *Stantonia*, Vietnam

## Abstract

The genus *Stantonia* Ashmead, 1904 (Hymenoptera, Braconidae, Orgilinae) is reviewed for Vietnam, China, Japan, and Russia. Six new species of the genus *Stantonia* are described and illustrated: *Stantonia brevicaudata* van Achterberg, **sp. n.**, *S. dickyyui* van Achterberg & Long, **sp. n.**, *S. granulata* Long & van Achterberg, **sp. n.**, *S. robustifemur* van Achterberg & Long, **sp. n.**, *S. stilpnosoma* Long & van Achterberg, **sp. n.**, and *S. vietnamica* van Achterberg, **sp. n.** A new subgenus (*Planitonia* **subg. n.**: type species *Stantonia robustifemur* van Achterberg & Long, **sp. n.**) is proposed for the species with a flat clypeus and face, and reduced vein r-m of the fore wing. Three species are newly recorded from Vietnam: *Stantonia gracilis* van Achterberg, 1987, *S. sumatrana* Enderlein, 1908, and *S. tianmushana* Chen, He & Ma, 2004. A key to species of *Stantonia* from Vietnam, China, Russia, and Japan is provided.

## Introduction

Members of the small subfamily Orgilinae Foerster, 1863 (Hymenoptera: Braconidae) are comparatively rarely collected and little is known about their biology (Shaw and Huddleston 1991). As far known, all species are solitary koinobiont endoparasitoids mainly in concealed lepidopteran larvae. The subfamily is subdivided into three tribes: van Achterberg, 1987 (Neotropical), Enderlein, 1905 (Neotropical (including Central America and southern U.S.A.), Afrotropical, Indo-Australian, NE Palaearctic) and Ashmead, 1900 (cosmopolitan). The tribe consists mainly of the genus *Stantonia* Ashmead, 1904, with 75 valid species of which 31 occur in the Oriental region (Braet and Quicke 2004). Four of the Oriental species intrude in the NE Palaearctic region and are included in the review; the only species known from Far East Russia may belong to an Oriental species but without having females available this remains still uncertain. The genus was revised by van Achterberg (1987; Indo-Australian spp.), Braet and Quicke (2004; worldwide), and Chen et al. (2004; for China). In this paper some new species are described, the interpretation of some species are corrected, and a new identification key for the species from Vietnam, China, Japan, and Russia is presented.

## Materials and methods

The specimens were mainly collected in Malaise traps, but a few by using a sweep net. The material was stored in 70% ethanol, prepared with the AXA method (van Achterberg 2009; van Achterberg et al. 2010) and glued on card points. Observations and descriptions were made with an Olympus SZX11 stereomicroscope and fluorescent lamps. Photographic images were made with an Olympus motorized stereomicroscope SZX12 with AnalySIS Extended Focal Imaging Software and processed with Adobe Photoshop CC, mostly to adjust the size and background. The photographs of the types deposited in Vietnam were made by KDL with a Digital microscope camera MVV3000 attached to the Olympus SZ61 binocular microscope connecting to a computer at IEBR.

*Morphology*. For terminology used in this paper, see van Achterberg (1988, 1993). Measurements are taken as indicated by van Achterberg (1988). Additional non-exclusive characters in the key are between brackets. For the identification of the subfamily Orgilinae, see van Achterberg (1993) and for the genera of Orgilinae, van Achterberg (1994).

**Material.** The examined specimens are kept in the parasitoid collections of Department of Insect Ecology (**IEBR**) at Hanoi, Vietnam; the Naturalis Biodiversity Center, (**RMNH**) at Leiden, The Netherlands; the Institute of Zoology, Chinese Academy of Sciences (**IZAS**) at Beijing, China; the Zoological Institute, Akademia NAUK (**ZISP**) at St. Petersburg, Russia; the Entomological Collection, Zoological Museum, Hokkaido University (**ECHU**) at Sapporo, Japan; School of Life Sciences, Northwest University (**NWUX**) at Xi’an, China, and the Senckenberg Deutsches Entomologisches Institut (**SDEI**) at Müncheberg, Germany.

Inside Vietnam, the distribution of the species is followed in order of provinces from north to south, and outside Vietnam, distribution of species follows in alphabetical order. An asterisk indicates a new record.

## Systematics

### Stantonia

Taxon classificationAnimaliaHymenopteraBraconidae

Ashmead, 1904

Figs 1 , 2 , 3 , 4–5 , 6–15 , 16 , 17 , 18–22 , 23 , 24–35 , 36 , 37 , 38–45 , 46 , 47–57 , 58 , 59 , 60–70 , 71 , 72, 73 , 74 , 75–80 , 81 , 82–92 , 93–96 , 97, 98 , 99 , 100–110 , 111 , 112 , 113–122 , 123

Stantonia Ashmead, 1904: 146; Shenefelt 1970: 266–268; van Achterberg 1987: 20–49; Braet and Quicke 2004: 1515–1582; Chen et al. 2004: 351–367, 531–533; Long and van Achterberg 2014: 408. Type species: Stantonia flava Ashmead, 1904 (by monotypy) [examined].
Mimagathis Enderlein, 1905: 450. Type species: Mimagathis ashmeadi Enderlein, 1905 (designated by Viereck 1914). Synonymised by Muesebeck (1970) [examined].
Bentonia van Achterberg, 1992: 339. Type species: Bentonia longicornis van Achterberg, 1992 (by original designation). Synonymised by Braet and Quicke (2004) [examined].

#### Diagnosis.

Antenna slender and 1.3–2.0 times longer than body, basal flagellar segments with medial constriction; scapus robust and strongly oblique apically (Figs 33, 45, 79); clypeus normal (but either convex or flattened) and its ventral margin almost straight; occipital carina lamelliform, reaching up to upper level of eyes (Figs 2, 33); malar suture present (Fig. 35, especially in most Indo-Australian spp.) or absent; length of mesosoma 1.2–1.4 times its height; prepectal carina complete, almost reaching anterior margin of mesopleuron; precoxal sulcus narrowly impressed and more or less crenulate (Figs 25, 61, 72); metapleuron not projecting forwards ventro-laterally (Fig. 83), metapleural flange present or absent; notauli complete, mainly smooth or completely crenulate; mesoscutum evenly short setose, finely punctulate, shiny, smooth or coriaceous; scutellar sulcus crenulate or smooth; propodeum convex to rather flat, smooth or coriaceous-granulate, with some rugae or with medial carinae anteriorly and with areola posteriorly; vein 1-M of fore wing straight; vein r-m of fore wing present and partly sclerotized (Figs 82, 94, 96), but completely absent or unsclerotized (Fig. 60) in subgenus Planitonia subg. n.; vein cu-a of fore wing antefurcal, (sub)interstitial or shortly postfurcal, (sub)vertical; vein 2-M of fore wing sclerotized basally; vein SR1 of fore wing straight; vein 1-SR+M of fore wing present, rarely absent; hind wing with 3 hamuli; outer side of hind tibia with some pegs apically, rarely obsolescent; middle leg very slender compared with hind leg (Figs 4, 17, 18, 58), more pronounced than in other genera of Orgilinae; length of first metasomal tergite 1.9–3.3 times its apical width, and its dorsal carinae absent (Figs 63, 85, 103, 116); second tergite smooth, granulate or coriaceous, without depressions; second tergite with sharp lateral crease; second metasomal suture straight (Fig. 64) or curved (Fig. 28); third (except base) and fourth tergites without sharp lateral crease (Fig. 23); ovipositor without notch or nodus; length of ovipositor sheath 0.15–0.7 times fore wing, but 1.0–1.4 times in *S. lutea* and *S. robustifemur*.

#### Biology.

Koinobiont endoparasitoids of Pyralidae and Tortricidae.

#### Distribution.

Mainly circumtropical, with some species in East Palaearctic region.

#### Notes.

The subgenus Planitonia subg. n. (with type species *Stantonia robustifemur* van Achterberg & Long, sp. n.) is proposed for the species with flat clypeus and face, reduced vein r-m of the fore wing and long ovipositor sheath (1.0–1.4 times as long as fore wing). Besides the type species described in this paper, *S. lutea* (Szépligeti, 1910) belongs to it. The subgenus is only known from the Oriental region and the biology is unknown. The name is derived from “planus” (Latin for flat, because of the flat clypeus) and the generic name *Stantonia*. Gender: feminine.

The genus *Sulorgilus* van Achterberg, 1994, is superficially similar and occurs in the treated area (Long and van Achterberg 2016). It has vein cu-a of hind wing approximately as long as vein 1-M (vein cu-a much shorter than vein 1-M in *Stantonia*), antenna of ♀ shortened (long) and its 15 subapical segments distinctly moniliform (non-moniliform and slender, but intermediate in *S. robustifemur* and *S. lutea*) and hind femur densely punctate (usually sparsely punctate or punctulate).

##### Key to species of the genus *Stantonia* Ashmead from Vietnam, China, Japan, and Russia

**Table:** 

1	Clypeus flat (Figs 66, 68); length of ovipositor sheath 1.1–1.4 times as long as fore wing; vein r-m of fore wing absent or largely so (Fig. 59); outer side of middle tibia with dense pegs (Fig. 59); face flattened medially; [basal half of hind tibia brownish yellow but with ivory basal ring; hind femur robust (Fig. 65) and ventrally slightly widened subbasally (Fig. 70), with satin sheen and micro-sculpture ventrally; propodeum anteriorly mostly granulate; length of first metasomal tergite 1.8–2.6 times its apical width; humeral plate partly dark brown]; subgenus Planitonia subg. n.	***S. robustifemur* van Achterberg & Long, sp. n.**
–	Clypeus convex (Figs 88, 90); length of ovipositor sheath 0.1–0.6 times as long as fore wing; vein r-m of fore wing partly pigmented and usually sclerotised (Figs 82, 94, 96); outer side of middle tibia at most with a row of pegs (Fig. 117); face slightly convex medially; subgenus Stantonia Ashmead, 1904	**2**
2	Anterior tentorial pits below lower level of eyes or near it (Fig. 19) and malar space comparatively long in lateral view (Fig. 21); anterior half of propodeum distinctly punctate-rugose; [epipleuron of second tergite with vague brownish patch (Fig. 17); second metasomal tergite largely or entirely yellowish; temple moderately punctate dorsally (Figs 20, 22); basal half of hind coxa reticulate-punctate dorsally]	***S. clappae* Kittel, 2016**
–	Anterior tentorial pits dorsally distinctly above lower level of eyes (Figs 79, 88, 118) and malar space comparatively short (Figs 76, 120); anterior half of propodeum usually punctulate and largely smooth, spaced punctate or granulate	**3**
3	Second metasomal suture curved and medial area behind suture convex (Figs 80); length of ovipositor sheath 0.4–0.6 times as long as fore wing (but unknown of *S. spasskensis*); length of first metasomal tergite 3.2–4.2 times its apical width	**4**
–	Second metasomal suture straight and medial area behind suture flat or nearly so (Figs 28, 64), **if** slightly curved (Fig. 91) then third tergite flat medio-anteriorly; length of ovipositor sheath 0.1–0.6 times as long as fore wing; length of first tergite 2.0–3.7 times its apical width	**9**
4	Mesosoma entirely or largely black (Figs 3, 112); submedially antenna with a white or ivory band contrasting with blackish basal third of antenna (Figs 3, 112); hind femur black or dark brown medially (Figs 3, 112); hind tarsus (except basitarsus basally and telotarsus) whitish or ivory; epipleuron of second metasomal tergite dark brown medially (Figs 3, 112)	**5**
-	Mesosoma entirely yellow or brownish yellow (Figs 23, 46, 123); antenna without a pale band, at most somewhat paler submedially (Figs 23, 123); hind femur brownish yellow medially (Figs 23, 36, 123); epipleuron of second tergite entirely yellowish (Fig. 23), but partly darkened in *S. xiangqianensis* (Fig. 123); [second tergite distinctly convex basally; hind tarsus largely ivory or dark brown; apex of first tergite brownish yellow; hind femur with smooth interspaces and shiny ventrally; humeral plate entirely yellowish]	**7**
5	Antenna of both sexes with 14–17 white or ivory segments submedially (Fig. 112); fore wing evenly infuscated (Fig. 113); propodeum and metapleuron black posteriorly; second metasomal epipleuron with distinctly isolated and well defined dark brown patch medially (Fig. 117); [first tergite distinctly narrowed behind spiracles (Fig. 116); tegulum dark brown; second metasomal tergite 1.7 times longer than wide basally]	***S. vietnamica* van Achterberg, sp. n.**
–	Antenna of both sexes with 6–13 white or ivory segments (Figs 3, 75); fore wing only apically infuscated (Figs 3, 75); propodeum and metapleuron yellowish brown or ivory posteriorly; second metasomal epipleuron with fuzzy dark brown area medially (Fig. 3)	**6**
6	Epipleuron of second metasomal tergite partly brownish yellow (Fig. 3); propodeum widely pale yellowish or yellowish brown posteriorly (Fig. 3)	***S. annulicornis* Enderlein, 1921**
–	Epipleuron of second metasomal tergite largely infuscated (Fig. 74); propodeum narrowly yellowish brown posteriorly (Figs 74, 77)	***S. spasskensis* Belokobylskij, 1993**
7	Hind tarsus (except basitarsus) dark brown (Figs 23, 29); hind femur slightly widened subapically (Fig. 23); hind coxa largely coarsely transverse striate (Fig. 25); transverse rugae of propodeum distinctly developed (Fig. 26)	***S. dickyyui* van Achterberg & Long, sp. n.**
–	Hind tarsus (except telotarsus) ivory (Figs 46, 52, 123); hind femur parallel-sided subapically (Figs 46, 123); hind coxa largely irregularly and densely finely rugose (Fig. 48); transverse rugae of propodeum weakly developed (Fig. 48)	**8**
8	Vein 3-SR+SR1 of fore wing approx. 2.9 times as long as vein r (Fig. 47); second–fourth metasomal tergites entirely yellowish brown and less compressed (Fig. 46); propodeum anteriorly largely smooth	***S. issikii* Watanabe, 1932**
–	Vein 3-SR+SR1 of fore wing 3.7–3.8 times as long as vein r (Fig. 123); second–fourth tergites posteriorly darkened (Fig. 123) and strongly compressed apically; propodeum anteriorly partly sculptured	***S. xiangqianensis* Chen, He & Ma, 2004**
9	Ventrally hind femur coarsely reticulate-rugose, densely sculptured, rather matt **and** ventrally apical 0.3–0.6 of femur black or dark brown (both sexes); mesopleuron ventrally and mesosternum often largely black; hind tarsus (except telotarsus and base of basitarsus) ivory, but fourth segment more or less dark brown; vertex largely dark brown or black; second epipleuron with large dark brown or brown patch; length of first metasomal tergite 2.0–2.8 times its apical width; [vertex rather coarsely and densely punctate]	**10**
-	Ventrally hind femur smooth and shiny, finely punctate or finely to moderately coriaceous-rugose or -rugulose and matt; **if** rugose or rugulose, then hind femur ventrally entirely yellowish or nearly so; mesopleuron ventrally and mesosternum yellowish brown; hind tarsus (except basitarsus) and fourth segment largely dark brown or ivory; vertex yellowish brown; area below precoxal suture finely punctate; second epipleuron entirely yellow and without dark patch, but with patch in *S. takeuchii*; length of first metasomal tergite 3.0–3.7 times its apical width, but 2.4–2.7 times in *S. sumatrana* and *S. brevicaudata*	**11**
10	Length of ovipositor sheath approx. 0.2 times as long as fore wing and 0.3 times length of metasoma (Fig. 2); length of first tergite 2.4–2.8 times its apical width; precoxal sulcus below crenulae usually coarsely punctate anteriorly; hind basitarsus pale yellow or white basally (Figs 1, 2), at most slightly infuscated; ventrally basal 0.2–0.4 of hind femur yellow; epipleuron of second tergite with nearly equilateral triangular patch (Figs 1, 2); entire antenna pale brown to dark brown, at most slightly paler submedially (Fig. 1); second metasomal tergite shiny and smooth	***S. angustata* van Achterberg, 1987**
–	Length of ovipositor sheath 0.4–0.5 times as long as fore wing and approximately as long as metasoma or slightly shorter (Fig. 36); length of first tergite 2.0–2.2 times its apical width; precoxal sulcus below crenulae sparsely punctulate or spaced punctate anteriorly; hind basitarsus blackish basally (Fig. 36); ventrally basal 0.4–0.6 of hind femur yellow; epipleuron of second tergite with elongate subtriangular patch (Fig. 36); basal half of antenna of ♀ dark brown basally and pale brownish or ivory apically, resulting in a pale submedial band (less clearly defined in ♂); second tergite rather matt and superficially granulate	***S. gracilis* van Achterberg, 1987**
11	Vein r-m omitted comparatively low from vein 2-SR, and petiole of second submarginal cell distinctly longer than wide (Figs 38, 96, 111); fore wing subhyaline apically (Figs 38, 111); hind femur matt ventrally; ovipositor sheath approximately half as long as metasoma (Fig. 111) or longer (Fig. 37)	**12**
-	Vein r-m of fore wing omitted near connection of vein r and 3-SR+SR1 and petiole of second submarginal cell at most slightly longer than wide (Figs 6, 82, 94); fore wing more or less darkened apically (Figs 6, 71, 72); **if** hardly so (Fig. 82) then hind femur shiny ventrally and ovipositor sheath distinctly shorter than half length of metasoma (Fig. 81)	**13**
12	Vein CU1b subequal to vein 3-CU1 (Figs 95, 111); dorso-apically hind coxa with transverse carinae; length of ovipositor sheath 0.25–0.32 times fore wing, approximately half as long as metasoma (Fig. 111); first metasomal tergite of ♀ distinctly narrowed behind spiracle and 3.0-3.6 times as long as its apical width; propodeum and first–second metasomal tergites largely smooth	***S. tianmushana* Chen, He & Ma, 2004**
-	Vein CU1b 0.3 times as long as vein 3-CU1 (Fig. 38); dorso-apically hind coxa without transverse carinae (Fig. 42); length of ovipositor sheath 0.5 times fore wing and somewhat shorter than metasoma (Fig. 37); first tergite of ♀ indistinctly narrowed behind spiracle (Fig. 41) and approximately twice as long as its apical width; propodeum and first–second metasomal tergites finely granulate (Figs 40, 41)	***S. granulata* Long & van Achterberg, sp. n.**
13	Length of ovipositor sheath 0.40–0.60 times as long as fore wing (Figs 58, 72, 99); length of first tergite 3.0–3.7 times its apical width (Fig. 103); [hind femur densely finely sculptured and rather matt ventrally]	**14**
–	Length of ovipositor sheath 0.10–0.25 times as long as fore wing (Figs 4, 16, 71, 81); length of first tergite 2.4–3.3 times its apical width (Figs 9, 85)	**16**
14	Antenna of ♀ with 8–13 white or ivory segments submedially (Fig. 58); vertex moderately punctate; middle lobe of mesoscutum yellowish brown medially; [lateral lobes of mesoscutum more or less dark brown; tegulum brownish yellow; base and apex of first tergite and base of second tergite yellowish brown]	***S. qui* Chen, He & Ma, 2004**
–	Antenna of ♀ yellowish brown submedially (Figs 72, 99); vertex coarsely punctate and interspaces approximately as wide as punctures or less (Fig. 107); middle lobe of mesoscutum dark brown medially (Fig. 102)	**15**
15	Inner half of humeral plate (Fig. 102), base and apex of first tergite and base of second tergite (Fig. 103) dark brown; hind coxa latero-apically and second epipleuron with dark brown patch (Fig. 99); [lateral lobes of mesoscutum more or less infuscate medially]	***S. takeuchii* (Watanabe, 1937)**
–	Humeral plate entirely, base and apex of first tergite and base of second tergite yellowish brown (Fig. 73); hind coxa and second epipleuron entirely yellowish brown (Fig. 72)	***S. sauteri* Watanabe, 1932**
16	Lobes of mesoscutum dark brown or infuscate medially (Figs 16, 71); antenna of ♀ 1.6–1.7 times as long as fore wing; length of ovipositor sheath 0.17–0.25 times as long as fore wing; frons often entirely dark brown or blackish medially; [basal half of antenna largely yellowish brown; apex of hind coxa more or less dark brown dorsally]	**17**
–	Lobes of mesoscutum brownish yellow medially (Figs 8, 97); antenna of ♀ 1.7–1.8 times as long as fore wing; length of ovipositor sheath 0.10–0.22 times as long as fore wing; frons more or less brownish yellow medially (Fig. 12)	**18**
17	Second and third hind tarsal segments ivory or white (Fig. 71); hind femur dull and densely micro-sculptured ventrally; tegulum dark brown or blackish; [propodeum with coarse transverse rugae]	***S. ruficornis* Enderlein, 1921**
–	Second and third hind tarsal segments dark brown (Fig. 16); hind femur shiny and largely smooth ventrally; tegulum brownish yellow	***S. chaoi* Chen, He & Ma, 2004**
18	First metasomal tergite of ♀ strongly shiny and 3.3–3.7 times as long as its apical width (Fig. 85); propodeum strongly shiny and with weak transverse rugae (Fig. 85); interspaces between punctures of ventral face of hind femur distinctly shiny; hind coxa strongly shiny dorsally (Fig. 83); length of ovipositor sheath 0.10–0.15 times fore wing; vertex punctulate and interspaces much wider than punctures (Fig. 89); [apex of first tergite and base of second tergite yellowish brown; second tergite of ♀ 1.6–1.7 times as long as wide; hind basitarsus (except apex) ivory or white and remainder dark brown]	***S. stilpnosoma* Long & van Achterberg, sp. n.**
–	First tergite of ♀ with satin sheen and 2.4–3.0 times as long as its apical width (Fig. 9); propodeum with satin sheen and with coarse transverse rugae (Fig. 8); interspaces between punctures of ventral face of hind femur micro-sculptured and rather matt; hind coxa with satin sheen dorsally (Fig. 7); length of ovipositor sheath 0.16–0.22 times fore wing; vertex moderately punctate, interspaces mostly as wide as punctures (Fig. 12)	**19**
19	Humeral plate partly brown or dark brown (Fig. 97); penultimate antennal segment of both sexes at least twice as long as wide (Fig. 98); occipital carina wider lamelliform; length of fore wing 4.7–7.5 mm	***S. sumatrana* Enderlein, 1908**
–	Humeral plate entirely pale yellowish or slightly brownish (Fig. 8); penultimate antennal segment of both sexes 1.5–1.7 times as long as wide (Fig. 5); occipital carina narrow lamelliform (Fig. 12); length of fore wing 4.2–4.6 mm	***S. brevicaudata* van Achterberg, sp. n.**

### Stantonia angustata

Taxon classificationAnimaliaHymenopteraBraconidae

van Achterberg, 1987

Figs 1 , 2

Stantonia angustata van Achterberg, 1987: 27–28; Chen et al. 2004: 354–356, 531; Long and van Achterberg 2014: 408.

#### Material.

1 ♂ (RMNH), “C. Vietnam: Thua Thien Hué, Phong Dién N.R., n[ea]r base-camp, 15 km W [of] Phong My, 80–120 m, 23.iii.–6.iv.2001, Mal. traps, C. v. Achterberg & R. de Vries, RMNH’01”; 1 ♂ (IEBR), “N. Vietnam: Hoa Binh, Pa Co Hang Kia N.R., 20°44'37"N, 104°56'20"E, 1046 m, 9–23.x.2009, Mal. tr. 5, C. v. Achterberg & R. de Vries, RMNH’09”; 1 ♀ + 2 ♂ (RMNH, IEBR), S. Vietnam: Dông Nai, Cát Tiên N.P., c. 100 m, 13–20.v.2007, Mal. traps, C. v. Achterberg & R. de Vries, RMNH’07”; 1 ♀, (IEBR), “Orgi.009”, “NW. Vietnam: Lai Chau, Phong Tho, Tam Duong, Lai Nhi Thang, 09.x.2004, KDLong; 1 ♀ (VNMN), “Orgi.036”, “NE. Vietnam: Ninh Binh, Cuc Phuong N.P., 7-9.v.2002, KDLong; 1 ♂ (RMNH), id., but 9.iv.–13.v.2007, Mai Phu Quy & Nguyen Thanh Manh; 1 ♂ (RMNH), “N. Vietnam: Ninh Binh, Cuc Phuong N.P., n[ea]r entrance, c. 225 m, 15.iv.–1.v.2000, Mal. Tr. II, Mai Phu Quy, RMNH’00”; 1 ♂ (RMNH), “S. Vietnam: Dak Lak, Chu Yang Sin N.P., n[ea]r dam, 740–940 m, 1–10.vi.2007, Mal. traps, C. v. Achterberg & R. de Vries, RMNH’07”; 1 ♀ (RMNH), “C. Vietnam: Ha Tinh, Vu Quang N.P., 18°19'47"N, 105°26'28"E, 66 m, 4.iii.–15.iv.2011, Mal. trap 9, C. v. Achterberg & R. de Vries, RMNH’11”; 1 ♀ (RMNH), id., but 18°17'46"N, 105°25'52"E, 106 m, 5.iii.–15.iv.2011, Mal. trap 12; 2 ♂ (IZAS), “[China:] Sichuan, Mt. Emei, 550-750 m, 19 & 20.v.1957”.

#### Diagnosis.

Antenna dark brown, but scapus and pedicellus partly pale; vertex rather coarsely and densely punctate and largely dark brown or black; anteriorly precoxal sulcus below crenulae coarsely punctate; mesosoma yellow with black spots; tegulum blackish; mesopleuron dark ventrally and rather coarsely punctate; propodeum rugose medially but anteriorly largely smooth; hind tarsus pale yellow or white but fourth and fifth segments more or less dark brown; ventrally hind femur coarsely reticulate-rugose, densely sculptured and rather matt; ventral apical 0.3-0.6 of femur black or dark brown (both sexes); hind femur 6 times longer than wide; ventrally basal 0.2–0.4 of hind femur yellow; epipleuron of second tergite with equilateral triangular dark brown patch; length of ovipositor sheath 0.18–0.24 times as long as fore wing and 0.3 times length of metasoma; length of fore wing 4.7–6.3 mm.

#### Variation.

**Figure 1.**
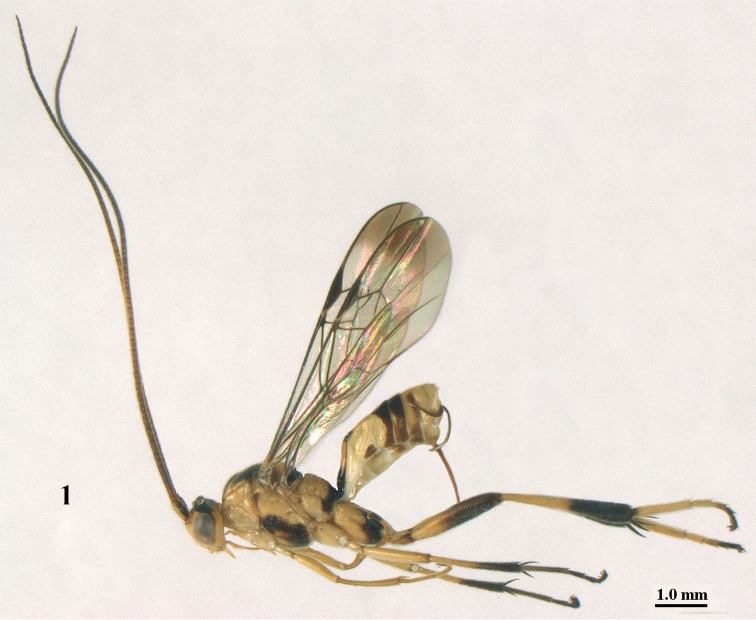
*Stantonia angustata* van Achterberg, ♀, Vietnam, Cát Tiên N.P., habitus, lateral aspect.

**Figure 2.**
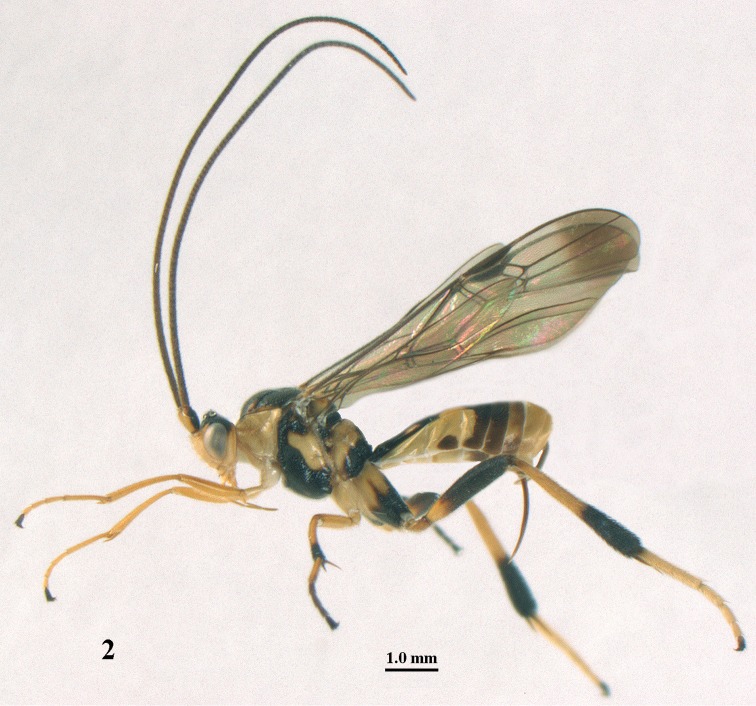
*Stantonia angustata* van Achterberg, ♀, Vietnam, Vu Quang N.P., habitus, lateral aspect.

Hind tibial spurs blackish (Cát Tiên N.P. and Cuc Phuong N.P.; Fig. 1), brown (Vu Quang N.P.; Fig. 2) or yellowish (Phong Dién N.R.); typical *S. angustata* have blackish spurs. Vietnamese specimens have hind tarsus (except dark telotarsus (Fig. 2) and sometimes (Fig. 1) fourth segment dark brown or brown) ivory or white; typical *S. angustata* have also third hind tarsal segment dark brown and second segment more or less infuscated.

#### Distribution.

China (Sichuan, Yunnan), Brunei, East and West Malaysia, Vietnam (Lai Chau; Ninh Binh, Cuc Phuong N.P. (Long and van Achterberg 2014); *Hoa Binh, Pa Co Hang Kia N.R.; *Thua Thien Hué, Phong Dién N.R.; *Dak Lak, Chu Yang Sin N.P.; *Dông Nai, Cát Tiên N.P.).

#### Notes.

If length of antenna 1.9 times fore wing, head blackish brown (except vertex); metasoma laterally blackish brown; second epipleuron with faint dark spot; length of body 3.8–5.3 mm, cf. *S. jacobsoni* van Achterberg, 1987.

### Stantonia annulicornis

Taxon classificationAnimaliaHymenopteraBraconidae

Enderlein, 1921

Fig. 3

Stantonia annulicornis Enderlein, 1921: 58; Shenefelt 1970: 267; van Achterberg 1987: 21; Chenet al. 2004: 531; Long and van Achterberg 2014: 408.
Stantonia spasskensis ; Long and van Achterberg 2014: 408.

#### Material.

1 ♀ (RMNH), “S. Vietnam: Dông Nai, Cát Tiên N.P., c. 100 m, 9.iv.–13.v.2007, Mal. traps, Mai Phu Quy & Nguyen Thanh Manh, RMNH’07”; 1 ♀ (RMNH), “S. Vietnam: Dak Lak, Chu Yang Sin N.P., n[ea]r dam, 740–940 m, 1–10.vi.2007, Mal. traps, C. v. Achterberg & R. de Vries, RMNH’07”; 1 ♀ (VNMN), ‘Orgi.039’ NE. Vietnam: Ninh Binh, Cuc Phuong NP, Bong forest, 13.v.2005, K.D. Long; 1 ♂ + 1 ♀ (IEBR), “Orgi 050 &051”, “C. Vietnam: Thua Thien-Hue, Nam Dong, MT 2–6.v.2005, N.Q. Truong; 1 ♀ (IEBR), “Orgi.068”, “NE Vietnam: Phu Tho, Tan Son, Lai Dong, MT, 21°13'N, 104°55'E, 180 m, 20.v.2011, K.D. Long; 1 ♂ (IEBR), “Orgi.003”, “NW. Vietnam: Hoa Binh, Yen Thuy, orchard, MT, 20°23'N, 105°36'E, 55 m, 20-30.iv.2002, K.D. Long”.

#### Diagnosis.

**Figure 3.**
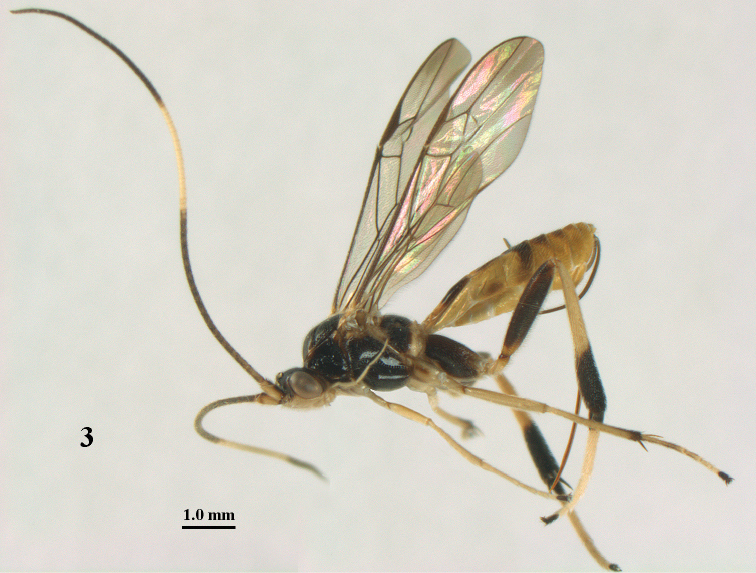
*Stantonia annulicornis* Enderlein, ♀, Vietnam, Cát Tiên N.P., habitus, lateral aspect.

Antenna of both sexes with band of 10–13 white or ivory segments (Fig. 3); mesosoma largely and telotarsi black or dark brown; tegulum pale brown or pale yellowish; metapleuron and propodeum posteriorly, and propodeum medially more or less yellowish brown or brown; fore wing only apically infuscated; hind tarsus (except base of basitarsus and telotarsus) whitish or ivory and conspicuously bristly setose; hind femur (except basally) and more or less middle coxa black, dark brown or brownish dorsally; ovipositor sheath 0.55–0.60 times as long as fore wing; epipleuron of second metasomal tergite entirely pale yellowish or with a faint brownish spot; length of fore wing 6–9 mm.

#### Distribution.

Myanmar, Vietnam (Phu Tho; Hoa Binh; Ninh Binh, Cuc Phuong N.P.; Thua Thien-Hue (Long and van Achterberg 2014); *Dak Lak, Chu Yang Sin N.P.; *Dông Nai, Cát Tiên N.P.).

#### Notes.

Holotype of *S. annulicornis* from Myanmar has the middle coxa largely black (mainly brown to black in Vietnamese specimens), the propodeum finely punctate (variable in Vietnamese specimens, but often largely smooth) and the tegulum pale yellowish (pale yellowish brown to brown in Vietnamese specimens).

### Stantonia brevicaudata

Taxon classificationAnimaliaHymenopteraBraconidae

van Achterberg sp. n.

http://zoobank.org/6D75C52B-C75F-464B-8317-4BF78333A6D6

Figs 4–5 , 6–15

#### Type material.

Holotype, ♀ (RMNH), “Vietnam: Ninh Thuân, Núi Chúa N.P., northeast part, Mal. traps, 90–150 m, 24–30.v.2007, C. v. Achterberg & R. de Vries, RMNH’07”. Paratype: 1 ♂ (RMNH), “S. Vietnam: Dông Nai, Cát Tiên N.P., c. 100 m, 13–20.v.2007, Mal. traps, C. v. Achterberg & R. de Vries, RMNH’07”.

#### Diagnosis.

**Figures 4–5.**
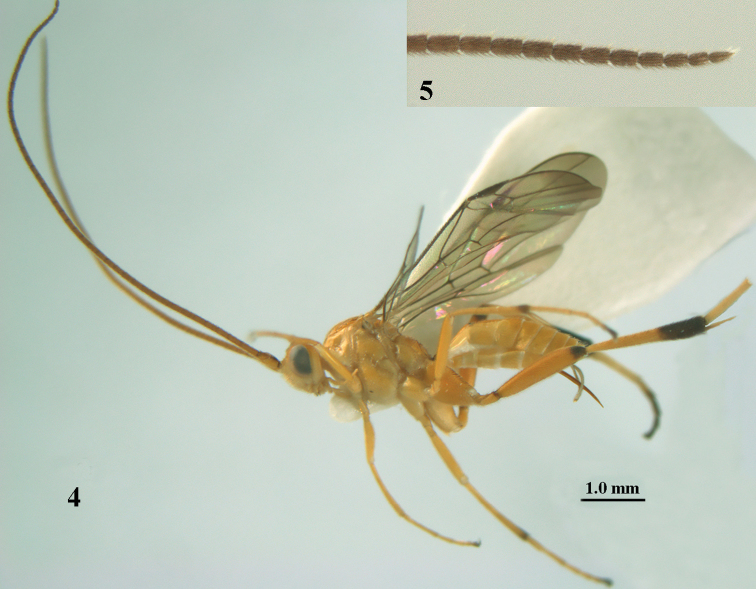
*Stantonia brevicaudata* sp. n., ♀, holotype. **4** habitus, lateral aspect **5** apex of antenna.

**Figures 6–15.**
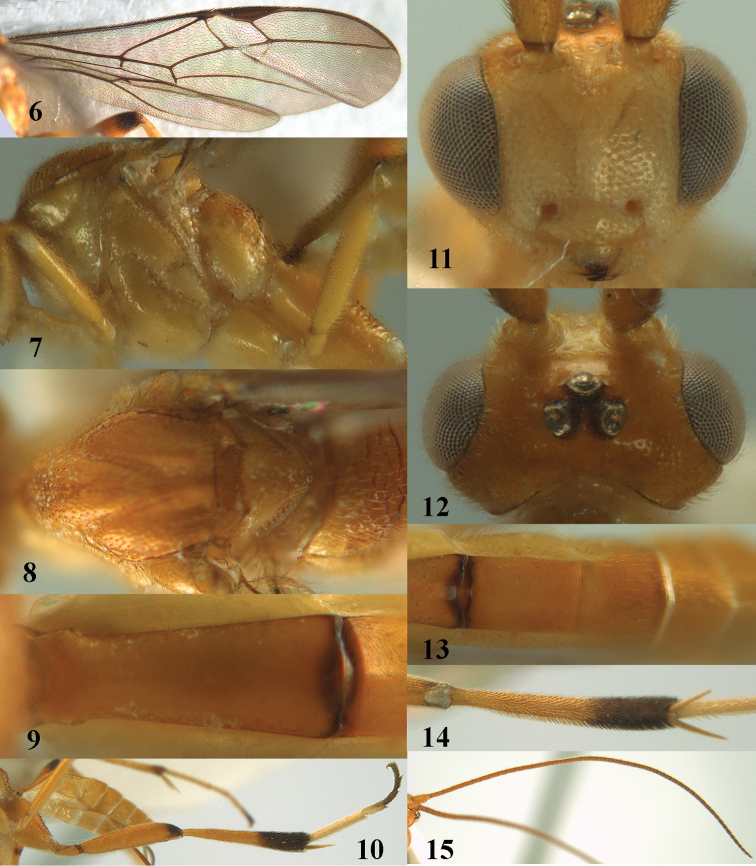
*Stantonia brevicaudata* sp. n., ♀, holotype. **6** wings **7** mesosoma, lateral aspect **8** mesosoma, dorsal aspect **9** first metasomal tergite, dorsal aspect **10** hind leg, lateral aspect **11** head, anterior aspect **12** head, dorsal aspect **13** second and third metasomal tergites, dorsal aspect **14** hind tibia, ventral aspect **15** antenna.

Antenna of ♀ 1.8 times as long as fore wing and largely brown (Fig. 4); apical antennal segments of both sexes 1.5–1.7 times as long as wide; vertex rather densely punctate and yellowish brown, but stemmaticum largely darkened (Fig. 12); mesosoma entirely pale brownish yellow; area below precoxal suture finely spaced punctate; tegulum and humeral plate entirely pale yellowish; propodeum with coarse transverse rugae; vein CU1b of fore wing strongly oblique and distinctly diverging from vein cu-a, short (Fig. 6); fore wing with apical part more or less infuscated (Fig. 6); ventrally hind femur mainly coriaceous, except some rugulae, matt (as outer side) and entirely yellowish brown; third and fourth segments of middle tarsus ivory or pale brown; apex of hind coxa yellowish brown dorsally; apex of hind basitarsus white or ivory (as more or less of second segment); third–fifth hind tarsal segment dark brown; first tergite of ♀ approx. 2.5 (of ♂ 2.6) times as long as its apical width and more or less widened apically; apex (and of ♂ also base) of first tergite and base of second tergite infuscate or dark brown (Fig. 9); second tergite largely smooth; second epipleuron entirely yellow; length of ovipositor sheath 0.2 times as long as fore wing and 0.3 times as long as metasoma; length of fore wing 4–5 mm.

The new species runs in the key by van Achterberg (1987) to *S. sumatrana* Enderlein, but differs by having the humeral plate entirely pale yellowish (partly infuscate or brown in *S. sumatrana*), penultimate antennal segments of both sexes 1.5–1.7 times as long as wide (at least twice as long as wide) and occipital carina narrow lamelliform (wider lamelliform).

#### Description.

Holotype, ♀. Body length 4.6 mm, fore wing length 4.4 mm, ovipositor sheath 0.7 mm.

*Head*. Antenna with 49 segments and 1.8 times as long as fore wing; middle antennal segments with distinct false division medially and 1.8 times as long as wide; third, fourth and penultimate antennal segments 3.6, 2.6 and 1.7 times as long as wide, respectively, and third segment 1.4 times as long as fourth segment; width of face equal to height of face and clypeus combined (Fig. 11); maxillary palp approximately as long as height of head; clypeus distinctly convex (Fig. 11); malar space 1.2 times as long as mandible width; distance between tentorial pits 1.8 times as long as distance between pit and eye margin; in anterior view length of eye 1.8 times as long as wide; in dorsal view length of eye 2.6 times as long as temple; POL:OD:OOL = 3:3:6; distance between anterior and lateral ocellus 0.7 times OD (Fig. 12); face remotely and rather coarsely punctate and medium-sized setae; vertex finely remotely punctate and directly behind stemmaticum depressed; temple matt and with indistinct micro-sculpture; occipital flange wide lamelliform.

*Mesosoma.* Length of mesosoma 1.3 times as long as high; pronotal side smooth dorsally and remainder sparsely finely punctate and medial sulcus anteriorly with few crenulae; notauli rather narrow and moderately crenulate (Fig. 8); mesoscutum and scutellum remotely and often rather coarsely punctate (Fig. 8); precoxal sulcus narrow and finely crenulate, but obsolescent anteriorly and posteriorly (Fig. 7), meso- and metapleuron sparsely finely punctate; propodeum rather shiny, with coarse transverse rugae (Fig. 8), but anteriorly and posteriorly mainly smooth.

*Wings*. Fore wing (Fig. 6): pterostigma 4.4 times as long as wide; r:2-SR:3-SR+SR1:r-m = 5:7:26:5; r issued submedially from pterostigma; r-m only submedially weakly sclerotized; cu-a slightly postfurcal (Fig. 6); basal half of CU1a largely sclerotized; CU1b: 3-CU1 = 5:9. Hind wing: M+CU:1-M: 1r-m = 5:14:1.

*Legs*. Ventrally hind femur mainly coriaceous, except some rugulae, matt (as outer side); length of femur, tibia and basitarsus of middle leg 7.4, 11.9 and 11.6 times as long as their width, respectively; inner and outer middle tibial spurs 0.55 and 0.40 times as long as basitarsus; length of femur, tibia and basitarsus of hind leg 5.0, 7.7 and 6.8 times their width, respectively; inner and outer hind tibial spurs 0.5 and 0.4 times as long as basitarsus, respectively.

*Metasoma*. First tergite gradually widened (Fig. 9), 2.5 times as long as its apical width, its surface largely smooth, rather dull and apically slightly micro-sculptured; second tergite smooth (except some punctures), elongate, 1.5 times longer than its basal width and rather dull; second suture straight and area behind rather flat; length of ovipositor sheath 0.17 times as long as fore wing and 0.3 times as long as metasoma (Fig. 4).

*Colour*. Yellowish brown dorsally and remainder (including tegulum and humeral plate) pale yellowish, but antenna (except scapus and pedicellus) and ovipositor sheath brown, apex of first tergite, base of second tergite, telotarsi, apex of hind femur, apex of middle tibia, apical 0.4 of hind tibia, third and fourth hind tarsal segments, dark brown; hind basitarsus and second tarsal segment ivory, but apex of latter slightly infuscated (Fig. 10); apex of fore wing moderately darkened and remainder subhyaline (Fig. 6); veins and pterostigma dark brown.

*Male*. Body length 4.5 mm, fore wing length 4.2 mm; length of first metasomal tergite 2.6 times its apical width.

#### Distribution.

Vietnam (Ha Tinh, Vu Quang N.P.; Ninh Thuân, Núi Chúa N.P.; Dak Lak, Chu Yang Sin N.P.; Dông Nai, Cát Tiên N.P.).

### Stantonia chaoi

Taxon classificationAnimaliaHymenopteraBraconidae

Chen, He & Ma, 2004

Fig. 16

Stantonia chaoi Chen, He & Ma, 2004: 356–358, 533; Long and van Achterberg 2014: 408.

#### Material.

1 ♀ (RMNH), “N. Vietnam: Ninh Binh, Cuc Phuong N.P., n[ea]r entrance, c. 225 m, 1–15.v.2000, Mal. tr. II, Mai Phu Quy, RMNH’00”; 2 ♀ (ZISP), N. Vietnam, Ba Vi, 70 km NW Hanoi, 400 m, forest; 1 ♂ (ZISP), N. Vietnam, Cao Phong, Ky Son, Ha Son Binh, forest; 1 ♀ (IEBR), “Orgi.035”, “NW Vietnam: Hoa Binh, Yen Thuy, 20°13'06"N, 105°34'11"E, 315m, 10–20.vi.2002, K.D. Long”; 2 ♂ (IEBR), “Orgi.072 & 075”, “NW Vietnam: Hoa Binh, Mai Chau, orchard, MT, 20°43'10.3"N, 104°59'47.0"E, 950 m, 1–10.v.2010, K.D. Long; 1 ♂ (IEBR), “Orgi.082”, “C Vietnam: Quang Nam, Dong Giang, P’Rao, 500-600 m 28.v.2006, HV Tru”; 1 ♀ (IEBR), “Orgi.084”, “NW Vietnam: Hoa Binh, Kim Boi, Thong Tien NR, MT, 20°39'24.7"N, 105°27'14.3"E, 200 m, 5–15.xi.2012, K.D. Long; 2 ♀ (VNMN), “Orgi.100 & 101” + 1 ♂ (VNMN). “Orgi.102”, “NE Vietnam: Tuyen Quang, Na Hang, Thanh Tuong, forest, MT, 22°19'01"N, 105°24'02"E, 162 m, 15.iii.2017, K.D. Long; 1 ♂ (IEBR), “Orgi.033”, “NC Vietnam, Ha Tinh, Huong Son, Rao An, forest, 200 m, 11.v.1998, K.D. Long”.

#### Diagnosis.

**Figure 16.**
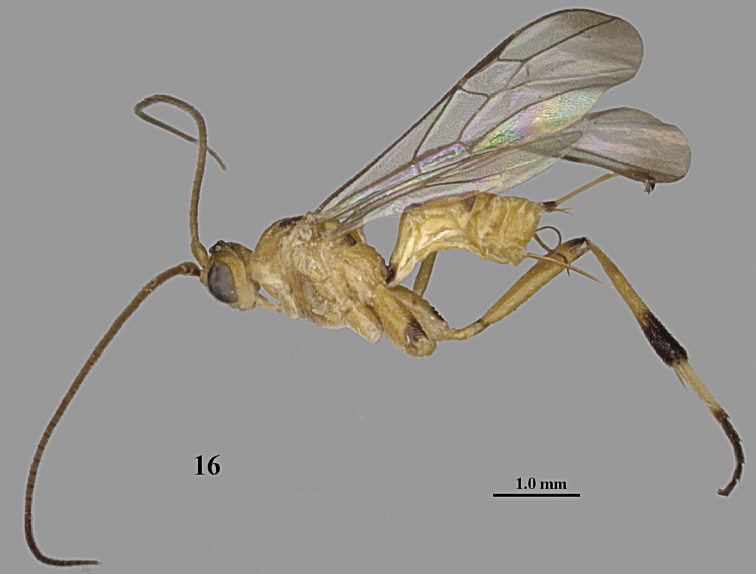
*Stantonia chaoi* Chen, He & Ma, ♀, holotype, habitus, lateral aspect. Photo: Jiachen Zhu.

Antenna of ♀ 1.6–1.7 times as long as fore wing and largely dark brown; frons with pair of dark brown spots posteriorly; vertex yellowish brown and strongly punctate, with interspaces approximately as wide as punctures or less; area below precoxal suture finely punctate; mesosoma yellowish brown, but lateral lobes of mesoscutum dark brown medially; mesoscutum and scutellum distinctly punctate; tegulum brownish yellow, but infuscate apically; propodeum rugose medially and remainder nearly smooth; fore wing infuscated apically; third segment of middle tarsus yellow or dark brown; outer side of hind femur rather shiny, parallel-sided and slender (Fig. 16); ventrally hind femur shiny and finely rugulose, and nearly entirely yellowish-brown; apex of hind basitarsus, third and fourth hind tarsal segments dark brown, similar to dark telotarsus; first tergite 2.7–3.0 times as long as wide apically; second epipleuron of metasoma entirely yellow; second metasomal suture straight; length of ovipositor sheath 0.17–0.21 times as long as fore wing and 0.2–0.3 times as long as metasoma; length of fore wing 4–6 mm.

#### Distribution.

China (Yunnan), Vietnam (Hoa Binh (Long and van Achterberg 2014); Tuyen Quang (Na Hang); Hoa Binh (Cao Phong, Kim Boi, Mai Chau, Yen Thuy); Ninh Binh (Cuc Phuong); Ha Tinh (Huong Son); Quang Nam (Dong Giang)).

### Stantonia clappae

Taxon classificationAnimaliaHymenopteraBraconidae

Kittel, 2016

Figs 17 , 18–22

Stantonia achterbergi Chen, He & Ma, (Sept.) 2004: 353–354, 531 (not S. achterbergi Braet & Quicke, (Feb.) 2004).
Stantonia clappae Kittel, 2016: 163 (replacement name).

#### Diagnosis.

**Figure 17.**
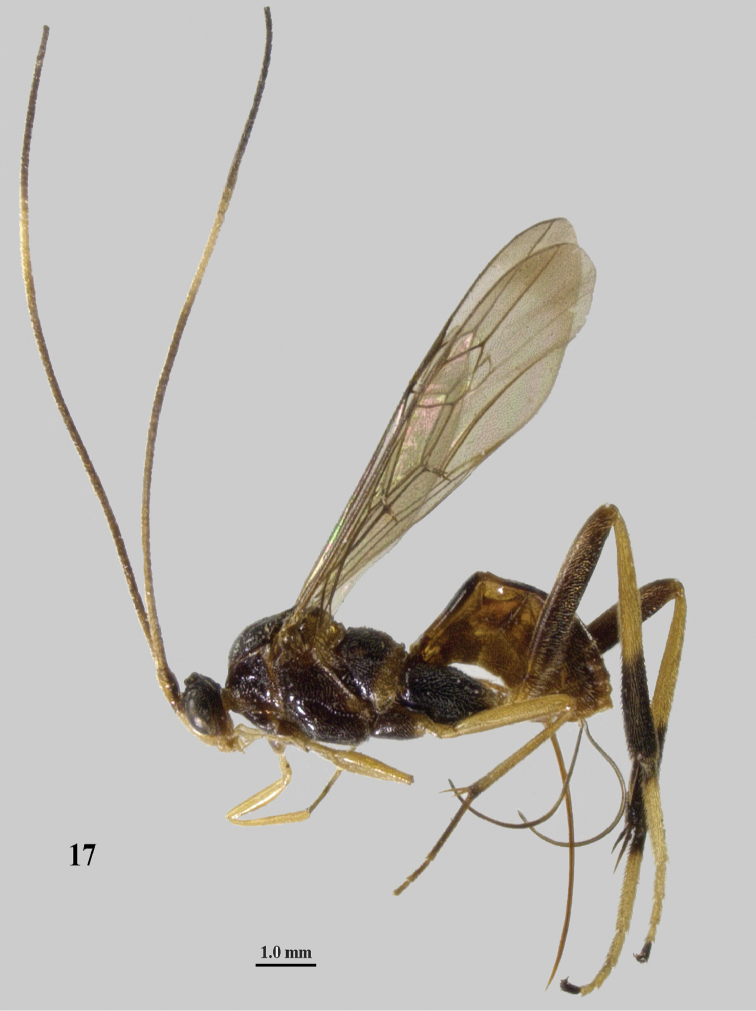
*Stantonia clappae* Kittel, ♀, paratype, China, Jilin, habitus, lateral aspect. Photo: Jiachen Zhu.

Apical half of antenna of both sexes with band of 6-9 ivory or white segments; anterior tentorial pits distinctly below lower level of eyes and malar space comparatively long (Fig. 19); tegulum brown or dark brown; mesosoma at least largely black or dark brown; anterior half of propodeum distinctly punctate; basal half of hind coxa reticulate-punctate dorsally hind femur (except basally) and more or less middle coxa black or dark brown dorsally; hind tarsus moderately bristly setose and (except basally and apically) whitish or ivory; infuscation of apex of fore wing occupies most of apex of fore wing (Fig. 17); first metasomal tergite approx. 4.3 times as long as wide; second metasomal tergite yellowish; epipleuron of second metasomal tergite with a dark brown or brown spot; length of ovipositor sheath 0.6 times as long as fore wing; length of fore wing 8–9 mm.

**Figures 18–22.**
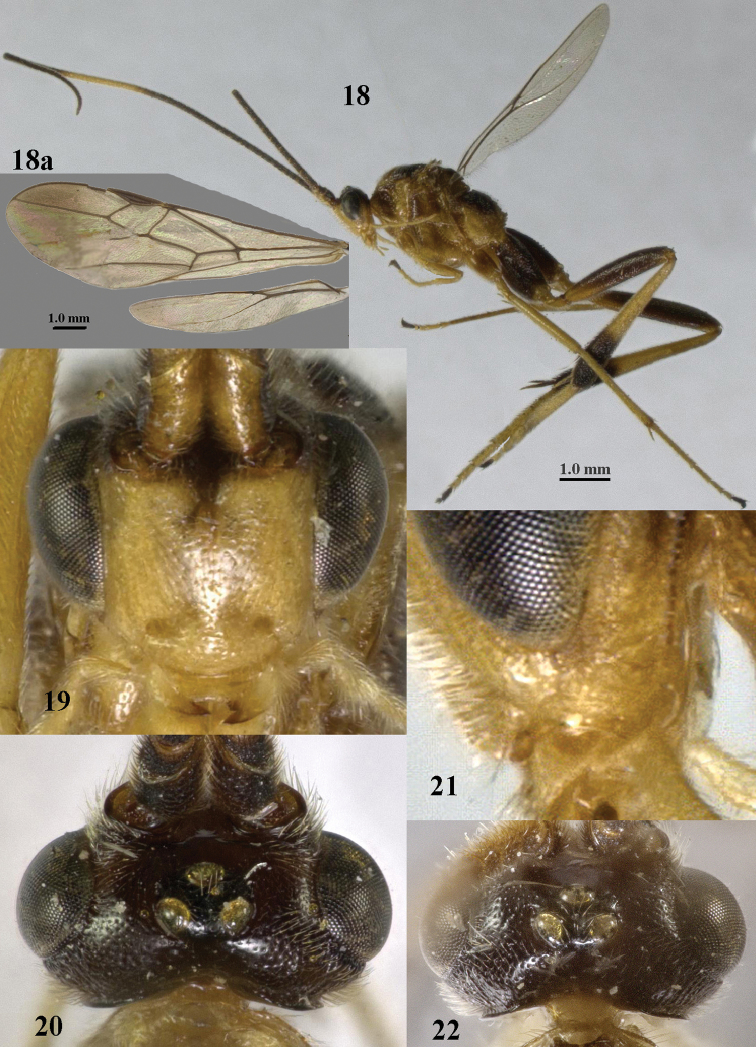
*Stantonia clappae* Kittel, ♀, holotype, China, Zhejiang, but 22 of paratype from Jilin. **18** habitus, lateral aspect (a= separated wings) **19** head, anterior aspect **20, 22** head, dorsal aspect **21** detail of malar space, lateral aspect. Photos: Jiachen Zhu.

#### Distribution.

China (Palaearctic: Jilin; Oriental: Guangdong, Zhejiang).

### Stantonia dickyyui

Taxon classificationAnimaliaHymenopteraBraconidae

van Achterberg & Long sp. n.

http://zoobank.org/86DF320F-FC9E-40C6-978B-E974ADD5F752

Figs 23 , 24–35

Stantonia xiangqianensis ; Long and van Achterberg 2014: 408.

#### Material.

Holotype, ♀ (RMNH), “N. Vietnam: Viet Try, n[ea]r Thanh Son, Thuong Cuu, 20°59'N, 105°8'E, 350–400 m, 11–16.x.1999, Malaise traps, R. de Vries, RMNH’99”. Paratypes (2 ♀ + 2 ♂): 1 ♀ (RMNH), “N. Vietnam: Ninh Binh, Cuc Phuong N.P., n[ea]r centre ([Mal. tr.] I), c. 225 m, 20.xii.1999–10.ii.2000, Mai Phu Quy, RMNH’00”; 1 ♂ (RMNH), id., but 15.iii.–14.ix.2000; 1 ♂ (RMNH), id., but 1.xi.–20.xii.2000; 2 ♀ (RMNH, IEBR), “C. Vietnam: Ha Tinh, Vu Quang N.P., 18°17'38"N, 105°25'25"E, 169 m, 24.ix.–5.x.2009, Taiw[an] tr[ap] 11, C. v. Achterberg & R. de Vries, RMNH’09”; 1 ♂ (VNMN), “Orgi.069”, “NC. Vietnam: Ha Tinh, Vu Quang N.P., forest, 6.x.2009, K.D. Long”.

#### Diagnosis.

**Figure 23.**
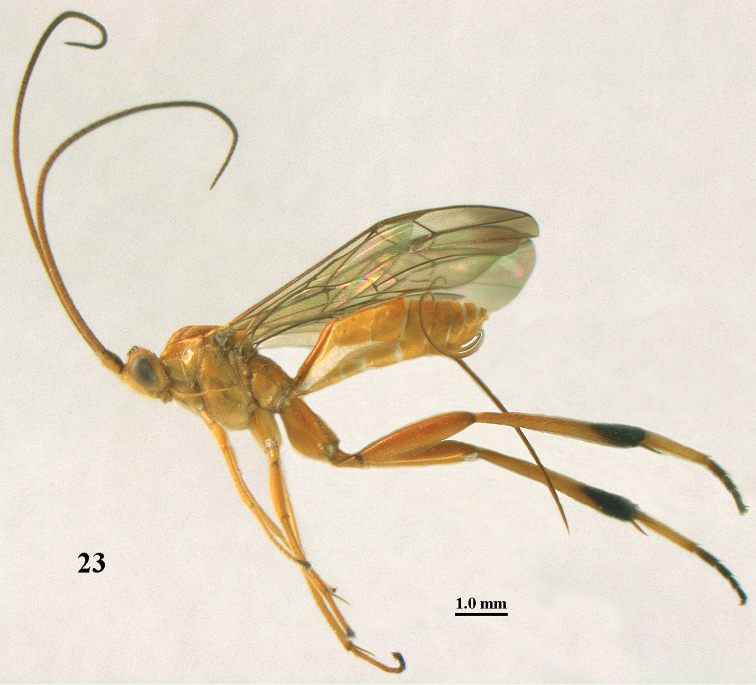
*Stantonia dickyyui* sp. n., ♀, holotype, habitus, lateral aspect.

**Figures 24–35.**
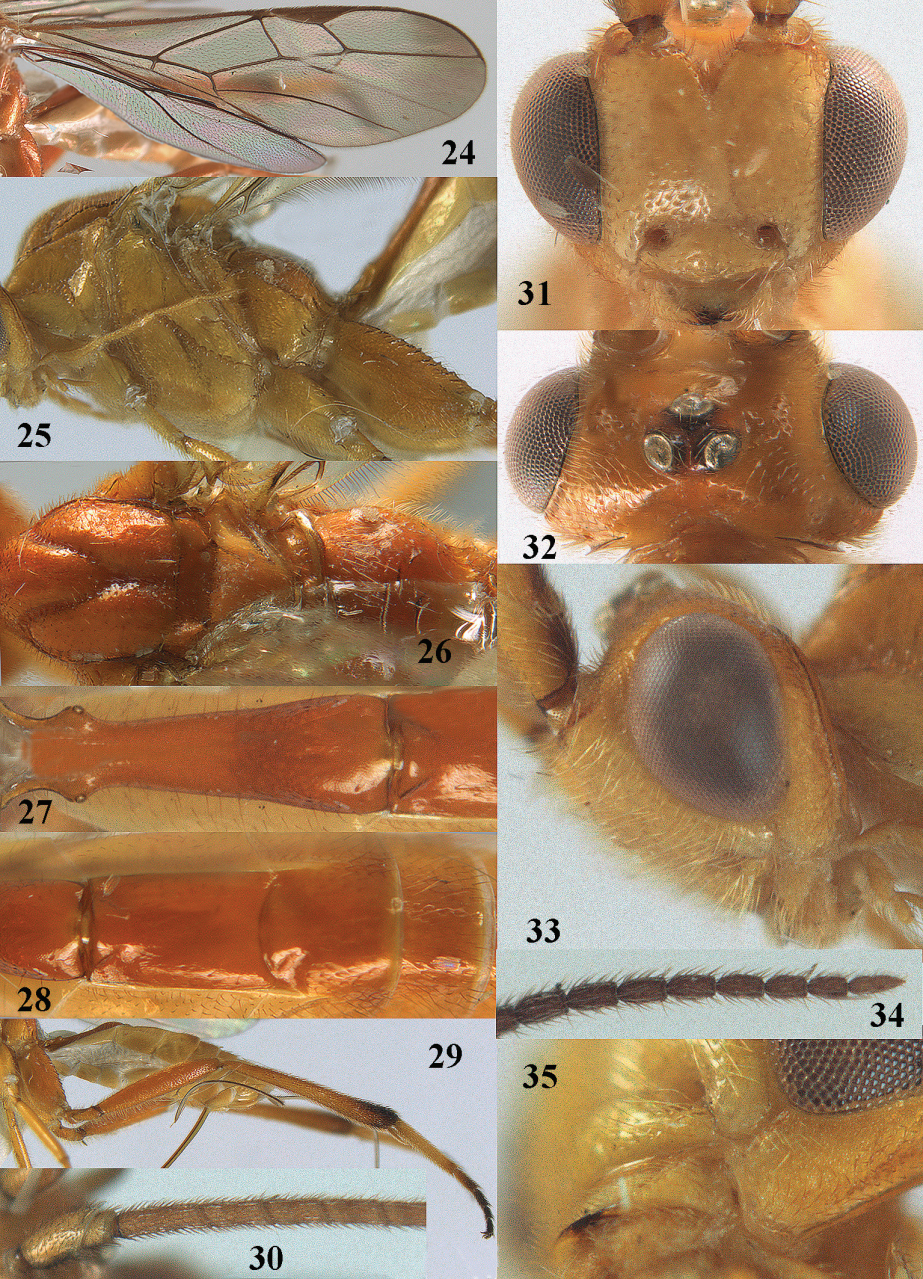
*Stantonia dickyyui* sp. n., ♀, holotype. **24** wings **25** mesosoma, lateral aspect **26** mesosoma, dorsal aspect **27** first metasomal tergite, dorsal aspect **28** second and third metasomal tergites, dorsal aspect **29** hind leg, lateral aspect **30** base of antenna, dorsal aspect **31** head, anterior aspect **32** head, dorsal aspect **33** head, lateral aspect **34** apex of antenna, dorsal aspect **35** mandible and malar space, lateral aspect.

Basal half of antenna yellowish, without ivory or white segments, its apical half, and outer side of scapus and pedicellus darkened; vertex finely spaced punctate and interspaces distinctly wider than punctures and yellowish brown; mesosoma entirely yellowish brown; inner half of humeral plate yellowish brown, remainder and tegulum brownish yellow; propodeum mainly smooth, medially with coarse transverse rugae (Fig. 26); fore wing moderately infuscated apically (Fig. 24); vein 3-SR+SR1 approx. 4 times as long as vein r; hind femur slightly widened subapically (Fig. 23), partly smooth and shiny ventrally, apically yellowish brown; hind tarsus (except largely ivory basitarsus) dark brown (Fig. 23); hind coxa largely coarsely transversely striate (Fig. 25); length of first metasomal tergite approx. 3.7 times its apical width, tergite strongly shiny; second epipleuron of metasoma without dark spot; apices of first and third metasomal tergites brownish yellow; second metasomal suture curved and medial area behind it convex; length of ovipositor sheath approx. 0.5 times as long as fore wing and somewhat longer than metasoma; length of fore wing 6–7 mm.

#### Description.

Holotype, ♀. Body length 6.8 mm, fore wing length 6.4 mm, ovipositor sheath 3.1 mm.

*Head*. Antenna with 58 segments and 1.7 times as long as fore wing; third, fourth and penultimate antennal segments 3.8, 2.4 and 2.1 times as long as wide, respectively, and third segment 1.6 times as long as fourth segment; width of face 0.9 times height of face and clypeus combined (Fig. 31); maxillary palp 1.6 times as long as height of head; clypeus convex dorsally and flattened ventrally, remotely finely punctate (Fig. 31); malar space as long as basal width of mandible; distance between large tentorial pits twice as long as distance between pit and eye margin; in anterior view length of eye 2.3 times as long as wide; in dorsal view length of eye 3.6 times as long as temple and temple directly narrowed behind eye; POL:OD:OOL = 8:10:21; distance between anterior and lateral ocellus 0.6 times OD (Fig. 32); face moderately convex, remotely punctulate, and with long setae; frons laterally and vertex remotely finely punctate (interspaces much wider than punctures), interspaces smooth and area directly behind stemmaticum depressed; frons medially smooth; stemmaticum strongly protruding; temple with satin sheen and mainly granulate, dorsally with some rugulae; occipital flange wide lamelliform.

*Mesosoma.* Length of mesosoma 1.3 times as long as high; pronotal side shiny and largely smooth except some superficial granulation ventrally and rather coarsely crenulate medial sulcus, subposteriorly absent and posteriorly narrowly crenulate; prepectal carina angulate and medium-sized; mesopleuron angulate ventrally; precoxal sulcus narrow and finely crenulate, complete and with wide flange posteriorly (Fig. 25), meso- and metapleuron remotely finely punctate, smooth interspaces much wider than punctures; notauli narrow and finely crenulate; mesoscutum finely punctate, with smooth interspaces much wider than width of punctures; scutellar sulcus smooth; scutellum remotely punctulate; propodeum shiny, mainly smooth but medially with some coarse rugae.

*Wings*. Fore wing (Fig. 24): pterostigma 4.4 times as long as wide; second submarginal cell petiolate; r:2-SR:3-SR+SR1:r-m = 10:12:39:6; r issued behind middle from pterostigma; r-m submedially distinctly sclerotized; cu-a slightly postfurcal (Fig. 25); basal 0.7 of CU1a more or less sclerotized; CU1b: 3-CU1 = 1:2; CU1b short. Hind wing: M+CU:1-M: 1r-m = 21:92:10.

*Legs*. Hind coxa with coarse curved rugae dorsally and shiny (Fig. 25); ventrally hind femur shiny and remotely finely punctate, long setose; length of femur, tibia and basitarsus of middle leg 7.4, 12.5 and 11.9 times as long as their width, respectively; inner and outer middle tibial spurs 0.50 and 0.35 times as long as basitarsus; length of femur, tibia and basitarsus of hind leg 5.3, 10.4 and 7.0 times their width, respectively; hind basitarsus rather erect setose; inner and outer hind tibial spurs 0.55 and 0.40 times as long as basitarsus, respectively.

*Metasoma*. First tergite distinctly narrowed behind spiracles (Fig. 27), 3.6 times as long as its apical width, its surface superficially finely granulate subapically and shiny; second tergite convex and smooth anteriorly, remainder smooth and shiny, 1.6 times longer than its basal width; second suture curved and medial area behind it convex; ovipositor sheath 0.49 times as long as fore wing and 0.9 times as long as metasoma (Fig. 23).

*Colour*. Yellowish brown; inner half of humeral plate yellowish brown, remainder of plate and tegulum brownish yellow; outer side of scapus and pedicellus, stemmaticum, pterostigma, hind tibial spurs, apex of hind basitarsus and base of second hind tarsal segment brown; apical half of antenna, remainder of hind tarsus, fore and middle telotarsi, fourth middle tarsal segment, apical 0.2 of hind tibia and ovipositor sheath dark brown; frons, face, clypeus, palpi, scapus and pedicellus ventrally, remainder of fore and middle legs, meso- and metasoma laterally and ventrally pale yellowish; apex of fore wing darkened and remainder subhyaline (Fig. 24); veins dark brown.

*Male*. Very similar to holotype; body length 6.6–6.8 mm, fore wing length 5.9–6.0 mm; antenna with 54(1), 56(1) segments and 1.9 times longer than fore wing; propodeum largely smooth and transverse rugae weakly or coarsely developed, length of first metasomal tergite 3.5–3.8 times its apical width; pterostigma and apical 0.3 of hind tibia dark brown.

#### Variation.

Female: length of body 6.2–6.8 mm and of fore wing 5.8–6.4 mm; antenna with 58(1), 57(2) segments; propodeum smooth and without distinct transverse rugae or with some weak or coarse rugae medially; vein cu-a of fore wing antefurcal or narrowly postfurcal; length of first tergite 3.2–3.6 times its apical width; length of ovipositor sheath 0.49–0.54 times fore wing.

#### Distribution.

Vietnam (Phu Tho (Viet Tri); Ninh Binh (Cuc Phuong); Ha Tinh (Vu Quang)).

#### Etymology.

Named after Dr Dicky Sick Ki Yu (Nepean, Canada) for creating Taxapad, the excellent and enormous database on parasitoid Hymenoptera. Nowadays, it is hardly imaginable to study successfully Braconidae without the help of this database.

### Stantonia gracilis

Taxon classificationAnimaliaHymenopteraBraconidae

van Achterberg, 1987

Fig. 36

Stantonia gracilis van Achterberg, 1987: 31–33; Braet and Quicke 2004: 1547.

#### Material.

2 ♀ (RMNH, IEBR), “S. Vietnam: Dông Nai, Cát Tiên N.P., c. 100 m, 9.iv.–13.v.2007, Mal. traps, Mai Phu Quy & Nguyen Thanh Manh RMNH’07”; 1 ♀ + 1 ♂ (RMNH), id., but 13–20.v.2007, C. v. Achterberg & R. de Vries.

#### Diagnosis.

**Figure 36.**
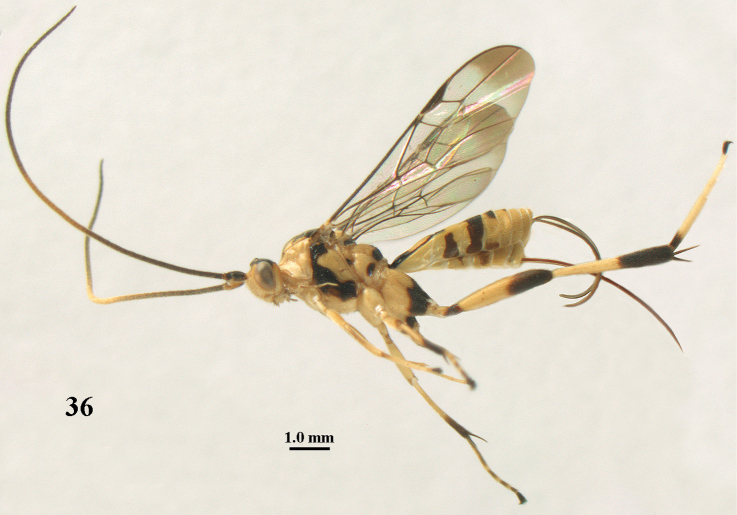
*Stantonia gracilis* van Achterberg, ♀, Vietnam, Cát Tiên N.P., habitus, lateral aspect.

Antenna of ♀ dark brown basally followed by pale brownish or ivory segments, resulting in a pale submedial band (Fig. 36; less clearly defined in ♂); vertex rather coarsely and densely punctate and largely dark brown or black; anteriorly precoxal sulcus below crenulae sparsely punctulate or spaced punctate; mesosoma yellow with black spots; tegulum blackish; mesopleuron yellowish ventrally and finely punctate; propodeum rugose medially but anteriorly largely smooth; hind tarsus pale yellow or white but base of basitarsus and telotarsus dark brown; ventrally hind femur coarsely reticulate-rugose, densely sculptured and rather matt; ventrally basal 0.6 of hind femur yellow; hind femur 6 times longer than wide; ventrally basal 0.2-0.4 of hind femur yellow; epipleuron of second tergite with elongate triangular dark brown patch; second metasomal tergite with weak triangular basal elevation length of ovipositor sheath 0.4–0.5 times as long as fore wing and approximately as long as metasoma or slightly shorter; length of fore wing 3.7–5.2 mm.

#### Distribution.

Indonesia (Sulawesi), Philippines (Luzon; Mindanao; Braet and Quicke 2004), *Vietnam (*Dông Nai, Cát Tiên N.P.). New record for Vietnam.

### Stantonia granulata

Taxon classificationAnimaliaHymenopteraBraconidae

Long & van Achterberg sp. n.

http://zoobank.org/0F5C009B-0E0E-4453-8C9F-9F82BC37FC3D

Figs 37 , 38–45

#### Type material.

Holotype, ♀ (VNMN), “Orgi.008”, “NC Vietnam: Huong Son, Ha Tinh, Son Tay, forest, 5–8.v.2004, TX Lam”.

#### Diagnosis.

Antenna of ♀ incomplete, with 37 segments remaining; basal two-thirds of remaining part of antenna yellow, apical third brown; tentorial pits at lower level of eyes (Fig. 43); malar space medium-sized; vertex finely punctate; anteriorly precoxal sulcus rugose-punctate, posterior area above precoxal sulcus finely granulate; propodeum finely granulate; hind basitarsus yellow basally and ivory apically; hind telotarsus dark brown and remainder of hind tarsus ivory; hind coxa yellow, rugose dorsally, granulate laterally; hind femur 6.6 times longer than wide and ventrally rugose-punctate; first metasomal tergite slightly narrowed behind spiracle; second metasomal suture straight; second tergite parallel-sided and granulate; ovipositor sheath 0.5 times as long as fore wing; length of fore wing 4.4 mm.

#### Description.

Holotype, ♀. Body length 4.6 mm, fore wing length 4.4 mm, ovipositor sheath 2.2 mm and exserted ovipositor 2.5 mm.

*Head*. Antenna incomplete, with 37 segments remaining; ventral length of scapus 2.3 times its maximum width; middle antennal segments 1.7–1.8 times as long as wide; third segment 1.2 times as long as fourth segment; width of face as long as height of face and clypeus combined (Fig. 43); maxillary palp nearly as long as height of head (30:31); clypeus distinctly convex (Fig. 43); malar space 1.75 times as long as mandible width (Fig. 45); distance between tentorial pits twice as long as distance between pit and eye margin; in anterior view length of eye 1.5 times as long as wide; in lateral view, width of eye 2.6 times temple; in dorsal view length of eye 2.8 times as long as temple; occipital carina broadly absent dorsally; ocelli large, POL:OD:OOL = 3:4:7; distance between anterior and lateral ocellus 0.5 times OD (Fig. 44); face largely punctate; vertex and temple finely punctate.

*Mesosoma.* Length of mesosoma 1.55 times as long as high; pronotal side crenulated medio-anteriorly; notauli deep, punctate, widened posteriorly; lobes of mesoscutum sparsely punctate; scutellar sulcus deep, 0.5 times as long as scutellum; precoxal sulcus short, punctate; anterior area above precoxal sulcus rugose-punctate; mesopleuron finely granulate posteriorly and ventrally; metapleuron granulate (Fig. 39); scutellum almost smooth (Fig. 40); propodeum finely granulate; propodeal spiracle rather large, 1.5 times as long as wide.

*Wings*. Fore wing (Fig. 38): pterostigma 4.8 times as long as wide; second submarginal cell petiolate; r:2-SR:3-SR+SR1:r-m = 11:12:48:8; vein r issued behind middle from pterostigma; cu-a slightly postfurcal, vein 1-CU1 nearly quadrate (Fig. 38); vein CU1b sclerotized, 0.4 times as long as 3-CU1 (Fig. 38). Hind wing: M+CU:1-M: 1r-m =16:29:3.

*Legs*. Hind coxa rugose dorsally, finely granulate laterally; length of femur and tibia of middle leg 6.6 and 10.4 times as long as their width, respectively; basitarsus of middle leg missing; length of femur, tibia and basitarsus of hind leg 4.4, 8.4 and 8.0 times their width, respectively; hind basitarsus 0.9 times as long as second-fifth segments; inner and outer hind tibial spurs 0.55 and 0.45 times as long as basitarsus, respectively.

**Figure 37.**
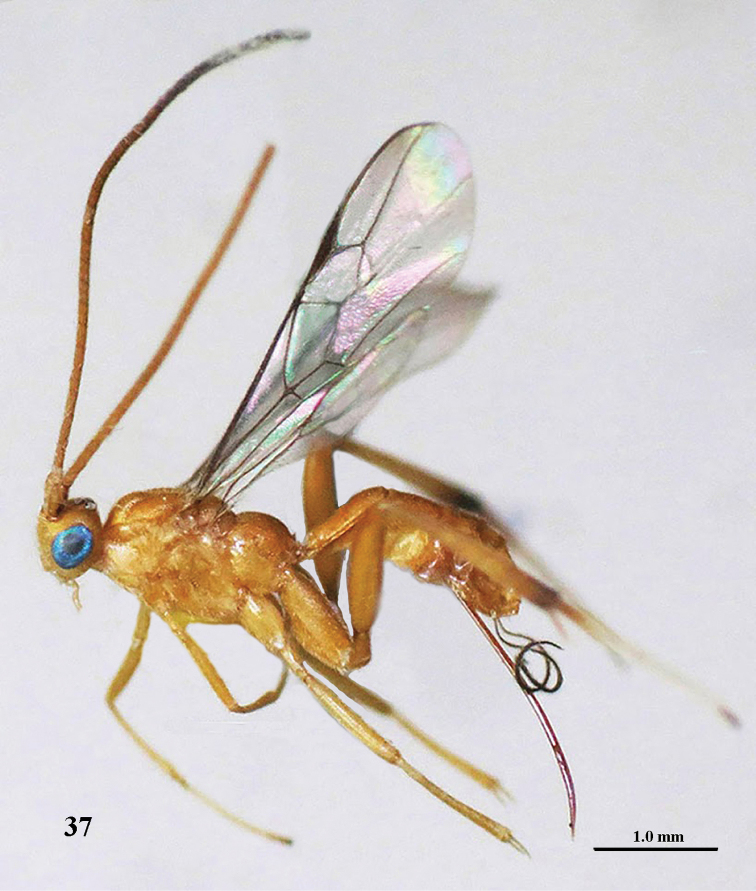
*Stantonia granulata* sp. n., ♀, holotype, habitus, lateral aspect.

*Metasoma*. First tergite slightly narrowed behind spiracles (Fig. 41), 2.1 times as long as its apical width, its surface finely granulate and 1.8 times as long as propodeum; second metasomal suture straight; second tergite parallel-sided, 1.15 times longer than third tergite; first and second metasomal tergites finely and densely granulate; third tergite granulate basally, punctate medially and smooth apically; ovipositor sheath 0.50 times fore wing and as long as metasoma; ovipositor thick (Fig. 37).

**Figures 38–45.**
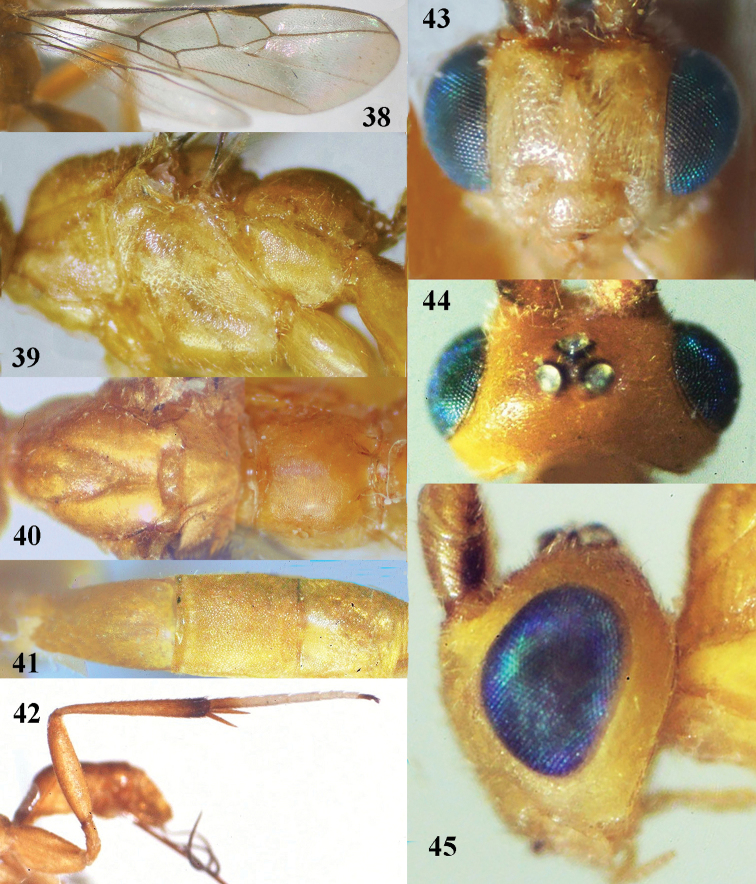
*Stantonia granulata* sp. n., ♀, holotype. **38** wings **39** mesosoma, lateral aspect **40** mesosoma, dorsal aspect **41** first–third metasomal tergites, dorsal aspect **42** hind leg, lateral aspect **43** head, anterior aspect **44** head, dorsal aspect **45** head, lateral aspect.

*Colour*. Yellow; antenna brownish yellow basally, dark brown apically; fore and middle legs yellow; hind leg yellow but telotarsus and apex of hind tibia dark brown, hind basitarsus yellow basally, remainder of hind tarsus white.

*Male.* Unknown; but two very similar males are present in VNMN (Orgi.086&087, NE Vietnam, Cao Bang; Trung Khanh, Cao Thang, MT 21-29.iv.2012, N.Q. Truong). They differ by having the body surface shinier and its sculpture less pronounced (propodeum rugulose-granulate, first–second metasomal tergites superficially granulate and sparsely punctate, and hind coxa more or less punctate laterally) and vein cu-a of fore wing interstitial.

#### Distribution.

NC Vietnam: Ha Tinh (Huong Son).

#### Etymology.

Named after the granulate hind coxae and propodeum; “granum” is Latin for “grain”.

### Stantonia issikii

Taxon classificationAnimaliaHymenopteraBraconidae

Watanabe, 1932

Figs 46 , 47–57

Stantonia issikii Watanabe, 1932: 187–188; Shenefelt 1970: 267; Braet and Quicke 2004: 1550–1551; Chen et al. 2004: 358–359, 532.

#### Type material.

Holotype, ♀ (ECHU), “Formosa [= Taiwan], Matsumura/ Kuraru, 21.iii.1926”, *Stantonia issikii* Watanabe, Type”.

#### Material.

1 ♀ (IZAS), China, Beijing, Shangfangshan National Forest Park, 400 m.

#### Diagnosis.

Antenna yellowish ventrally, only dorsally and apically darkened; vertex finely spaced punctate and interspaces distinctly wider than punctures and yellowish brown; mesosoma entirely yellowish brown; inner half of humeral plate dark brown, remainder and tegulum yellowish brown; propodeum medio-anteriorly smooth; fore wing moderately infuscated apically; vein 3-SR+SR1 approx. 3 times as long as vein r; hind femur partly smooth and shiny ventrally, slender and apically yellowish brown; hind tarsus (except telotarsus) ivory or white; length of first metasomal tergite approx. 3.7 times its apical width; second epipleuron of metasoma without dark spot; apices of first and third metasomal tergites brownish yellow; length of ovipositor sheath 0.5–0.6 times as long as fore wing and somewhat longer than metasoma; length of fore wing approximately 8 mm.

Very similar to *S. xiangqianensis* as indicated in the original description, but differs mainly by small colour differences and the relative length of vein r of the fore wing. The variation of these characters is unknown for both species and only large series may prove the validity of *S. xiangqianensis*.

#### Description.

Holotype, ♀. Body length 7.8 mm, fore wing length 8.2 mm, ovipositor sheath missing, exserted ovipositor 5.5 mm.

*Head*. Antenna broken; third and fourth antennal segments 3.2 and 2.7 times as long as wide, respectively, and third segment 1.2 times as long as fourth segment; width of face 0.9 times height of face and clypeus combined (Fig. 54); maxillary palp 1.6 times as long as height of head; clypeus distinctly convex (Fig. 54); malar space 1.2 times as long as mandible width; distance between large tentorial pits twice as long as distance between pit and eye margin; in anterior view length of eye 2.7 times as long as wide; in dorsal view length of eye 2.4 times as long as temple; POL:OD:OOL = 9:10:17; distance between anterior and lateral ocellus 0.6 times OD (Fig. 55); face remotely and moderately punctate and long setae; vertex remotely punctate, wide interspaces smooth and area directly behind stemmaticum depressed; temple with satin sheen and with mainly coriaceous; occipital flange wide lamelliform.

*Mesosoma.* Length of mesosoma 1.4 times as long as high; pronotal side largely smooth (with few punctures near dorsal rim) and medial sulcus coarsely and widely crenulate anteriorly, subposteriorly with two crenulate branches and posteriorly finely crenulate; precoxal sulcus narrow and finely crenulate, complete and with wide flange posteriorly (Fig. 48), mesopleuron remotely finely punctate; metapleuron moderately punctate; notauli rather narrow and moderately crenulate; mesoscutum and scutellum remotely and moderately punctate (Fig. 49); propodeum rather shiny, anteriorly smooth, posteriorly punctate and with some short transverse rugae medially and sublaterally.

*Wings*. Fore wing (Fig. 47): pterostigma 3.6 times as long as wide; second submarginal cell petiolate; r:2-SR:3-SR+SR1:r-m = 20:23:58:13; r issued behind middle from pterostigma; r-m submedially distinctly sclerotized; cu-a interstitial (Fig. 47); basal 0.7 of CU1a sclerotized; CU1b: 3-CU1 = 3:5. Hind wing: M+CU:1-M: 1r-m = 23:82:10.

*Legs*. Hind coxa largely and densely rugose dorsally, only posteriorly transversely striate; ventrally hind femur shiny, basally rugulose and apically largely smooth; length of femur, tibia and basitarsus of middle leg 7.0, 12.6 and 12.4 times as long as their width, respectively; inner and outer middle tibial spurs 0.40 and 0.35 times as long as basitarsus; length of femur, tibia and basitarsus of hind leg 5.2, 8.5 and 6.8 times their width, respectively; hind basitarsus rather adpressed; inner and outer hind tibial spurs 0.40 and 0.35 times as long as basitarsus, respectively.

**Figure 46.**
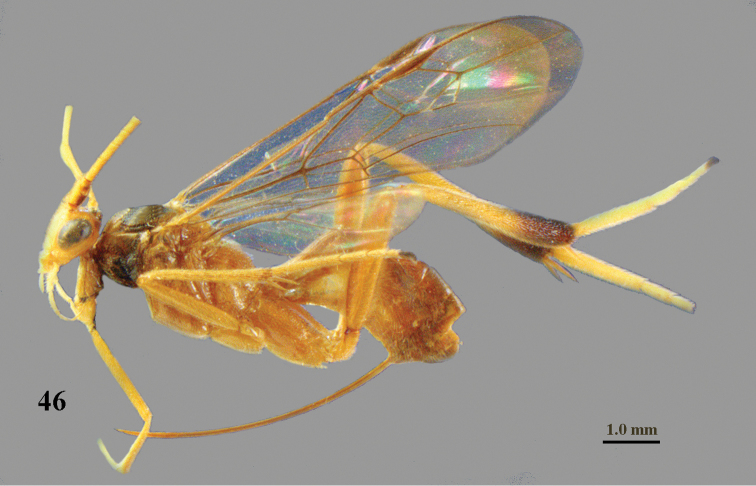
*Stantonia issikii* Watanabe, ♀, holotype, habitus, lateral aspect.

*Metasoma*. First tergite slightly narrowed behind spiracles (Fig. 50), 3.7 times as long as its apical width, its surface smooth and shiny; second tergite convex anteriorly, smooth (except some punctures), elongate, 1.8 times longer than its basal width and shiny; second suture curved and medial area behind it convex; ovipositor sheath missing, considering length of ovipositor approx. 0.6 times as long as fore wing and approximately as long as metasoma (Fig. 46).

**Figures 47–57.**
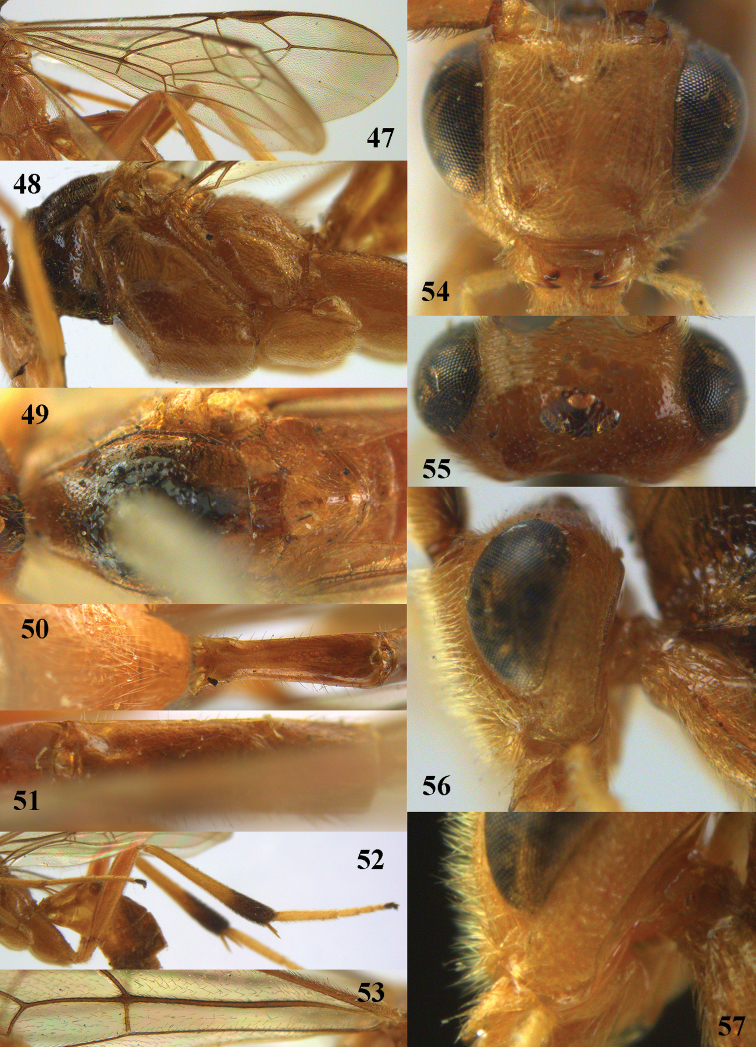
*Stantonia issikii* Watanabe, ♀, holotype. **47** fore wing **48** mesosoma, lateral aspect **49** mesosoma, dorsal aspect **50** propodeum and first metasomal tergite, dorsal aspect **51** second and third metasomal tergites, dorsal aspect **52** hind leg, lateral aspect **53** detail of submedial and first subdiscal cells of fore wing **54** head, anterior aspect **55** head, dorsal aspect **56** head, lateral aspect **57** occipital flange, postero-lateral aspect.

*Colour*. Yellowish brown; inner half of humeral plate dark brown, remainder of plate, tegulum and tibial spurs yellowish brown; basal segments of antenna (except scapus and pedicellus) dorsally dark brown and ventrally brownish yellow; outer side of scapus and pedicellus partly dark brown; face, clypeus, palpi and hind tibia (except apical third) rather pale yellowish; stemmaticum dark brown; apical third of hind tibia and telotarsi dark brown; remainder of hind tarsus ivory (Fig. 52); apex of fore wing moderately darkened and remainder subhyaline (Fig. 47); veins and pterostigma dark brown.

#### Distribution.

China (*Beijing (Shangfangshan N.F.P.), Zhejiang, Hunan, Taiwan).

#### Notes.

This species was reported from Papua New Guinea by Braet and Quicke (2004) with a question mark, but this concerns another species. The holotype differs by having distinctly rugose hind coxa (Fig. 48) and the fore wing is distinctly infuscated apically (Fig. 47).

### Stantonia qui

Taxon classificationAnimaliaHymenopteraBraconidae

Chen, He & Ma, 2004

Fig. 58

Stantonia qui Chen, He & Ma, 2004: 359–361, 531.

#### Diagnosis.

**Figure 58.**
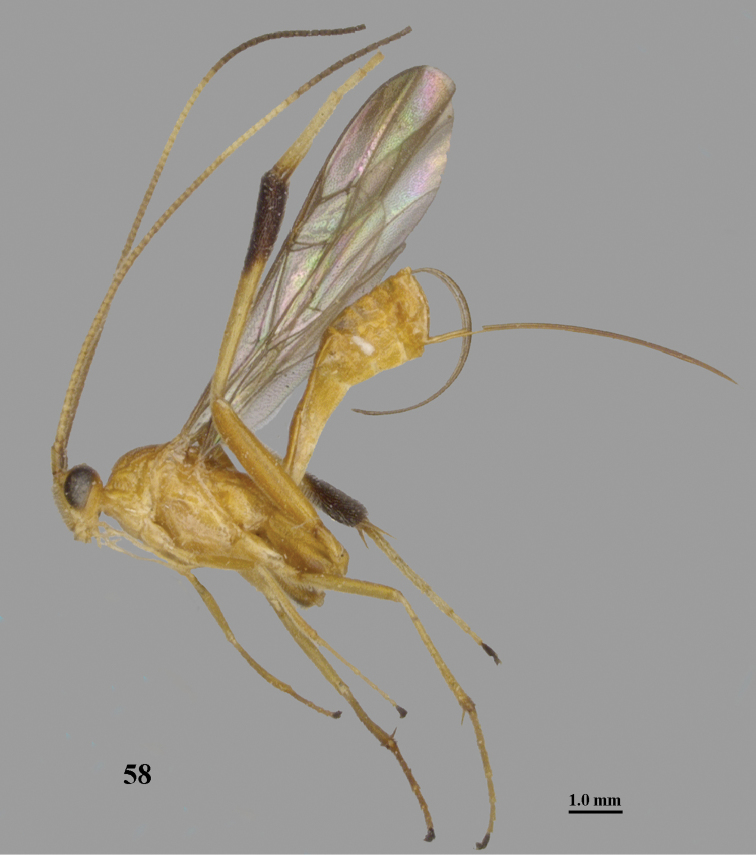
*Stantonia qui* Chen, He & Ma, ♀, holotype, habitus, lateral aspect. Photo: Jiachen Zhu.

Antenna with a submedial band consisting of 8–13 white or ivory segments (Fig. 58); face transversely punctate-rugose; vertex spaced punctate, interspaces wider than punctures; middle and lateral lobes of mesoscutum yellowish brown medially; tegulum brownish yellow; only apical half of marginal cell of fore wing infuscated; hind femur largely brownish yellow, at most its apical 0.3 dark brown; hind femur shiny and finely sculptured basally; middle and hind coxa pale yellowish; first metasomal tergite approx. 3.7 times as long as its apical width; metasoma dark yellowish brown; epipleuron of second metasomal tergite entirely yellowish brown (Fig. 58); length of ovipositor sheath approx. 0.5 times as long as fore wing; length of fore wing approx. 7 mm.

Similar to *S. magnifica* van Achterberg, 1987, from Indonesia and Malaysia, but *S. magnifica* differs by having the vertex largely smooth; the face finely punctate; the wing membrane dark brown up to apical 0.7 of the marginal cell; the hind coxa largely yellow or orange brown and the mesosoma entirely dark brown or black (Chen et al. 2004).

#### Distribution.

China (Guangdong, Zhejiang).

### Stantonia robustifemur

Taxon classificationAnimaliaHymenopteraBraconidae

van Achterberg & Long sp. n.

http://zoobank.org/402C8998-6372-45AF-82CC-1ED0D8DD4A6B

Figs 59 , 60–70

Stantonia sp. A Braet & Quicke, 2004: 1522.

#### Type material.

Holotype, ♀ (RMNH), “S. Vietnam: Dông Nai, Cát Tiên N.P., c. 100 m, 13–20.v.2007, Mal. traps, C. v. Achterberg & R. de Vries, RMNH’07”. Paratypes (5 ♀ + 1 ♂): 2 ♀ + 1 ♂ (RMNH, IEBR), same data as holotype; 1 ♀ (RMNH), id., but Bird trail, Mal trap[s] 30–35, 15–20.v.2007; 1 ♂ (IEBR), same data, but 9.iv.–13.v.2007, Mai Phu Quy & Nguyen Thanh Manh; 1 ♀ + 1 ♂ (VNMN), “Orgi.078 & 079”, “S Vietnam: Dong Nai, Cat Tien N.P., MT 11-25.iv.20 07, M.P. Quy, N.T. Manh”; 1 ♀ (RMNH), “N. Vietnam: Ninh Binh, Cuc Phuong N.P., n[ea]r entrance, c. 225 m, 15.iv.–1.v.2000, Mal. tr. II, Mai Phu Quy, RMNH’00”; 1 ♂ (RMNH), “S. Vietnam: Dak Lak, Chu Yang Sin N.P., Krong K’Mar, 740–900 m, 2–10.vi.2007, Mal. traps, C. v. Achterberg & R. de Vries, RMNH’07”. Excluded from type series: 1 ♀ (VNMN), “Orgi.006”, “NW Vietnam: Hoa Binh, Yen Thuy, orchard, MT 20°23'N, 105°36'E 55 m, 01-10.viii.2003, K.D. Long”.

#### Diagnosis.

Antenna without a pale band, its basal two-thirds brownish yellow and apical third dark brown, 1.3 times as long as fore wing and subapical segments approximately 1.5 times longer than wide; clypeus flat and rather long (Fig. 66); length of malar space 1.3–1.5 times basal width of mandible; tegulum brownish yellow; humeral plate partly dark brown; mesosoma entirely brownish yellow; propodeum anteriorly mostly granulate; vein r-m of fore wing absent or largely so (Fig. 60); hind femur robust and slightly widened subbasally (Fig. 65), ventrally with satin sheen and micro-sculpture; basal ring of hind tibia and hind tarsus ivory, except dark brown telotarsus and base of basitarsus; length of first tergite 2.0–2.6 times as long as wide apically; epipleuron of second tergite entirely yellow; second tergite rather matt and finely granulate; length of ovipositor sheath 1.1–1.4 times as long as fore wing; length of fore wing 4–5 mm.

The new species runs in the key by van Achterberg (1987) to *S. lutea* (Szépligeti, 1910) if the colour of the hind tarsus is not used, because of the long ovipositor sheath (1.0–1.4 times as long as fore wing), antenna 1.3 times as long as fore wing, yellowish tegulum and mesosoma, reduced vein r-m of fore wing, largely granulate propodeum, coriaceous-granulate first–third tergites, flat clypeus, ivory basal ring of hind tibia and dark brown apex of third tergite. It differs by the white or ivory third–fourth hind tarsal segments (dark brown in *S. lutea*), hind femur robust (normal), hind tibia without dark subbasal ring (present), and propodeum with few coarse transverse rugae in posterior half (entirely granulate or with rather weak transverse rugae).

#### Description.

Holotype, ♀. Body length 6.1 mm, fore wing length 4.9 mm, ovipositor sheath 5.3 mm.

*Head*. Antenna with 41 segments and 1.3 times as long as fore wing; middle antennal segments with distinct false division medially and twice as long as wide; third, fourth and penultimate antennal segments 3.0, 2.7 and 1.4 times as long as wide, respectively, and third segment 1.1 times as long as fourth segment; width of face equal to height of face and clypeus combined (Fig. 66); maxillary palp approximately as long as height of head; malar space 1.3 times as long as mandible width; distance between tentorial pits 1.7 times as long as distance between pit and eye margin; in anterior view length of eye 2.2 times as long as wide; in dorsal view length of eye 3.2 times as long as temple; POL:OD:OOL = 7:5:8; distance between anterior and lateral ocellus 0.6 times OD (Fig. 67); face remotely and rather coarsely punctate and medium-sized setae; vertex finely remotely punctate, with large smooth interspaces and gradually lowered behind stemmaticum; temple matt and finely coriaceous; occipital flange medium-sized lamelliform (Fig. 68).

*Mesosoma.* Length of mesosoma 1.3 times as long as high; pronotal side smooth above oblique and anteriorly crenulate medial sulcus, sparsely finely punctate posteriorly and ventrally superficially coriaceous; notauli complete and posteriorly moderately crenulate (Fig. 62); mesoscutum and scutellum remotely and finely crenulate but mesoscutum posteriorly rather coarsely punctate (Fig. 62); precoxal sulcus narrow and finely crenulate, but obsolescent anteriorly (Fig. 61), meso- and metapleuron sparsely finely punctate, mesopleuron with some crenulae anteriorly; propodeum rather matt and granulate, with few coarse transverse rugae posteriorly (Fig. 62).

*Wings*. Fore wing (Fig. 60): first discal cell distinctly truncate dorsally; pterostigma 4.4 times as long as wide; r-m absent; r:2-SR:3-SR+SR1 = 10:16:51; r issued behind middle from pterostigma; cu-a interstitial (Fig. 35); basal half of CU1a largely sclerotized; CU1b: 3-CU1 = 2:7. Hind wing: M+CU:1-M: 1r-m = 11:27:5; R1 with three distinct hamuli.

*Legs*. Hind coxa mainly coriaceous, postero-dorsally with rugulae; ventrally hind femur mainly coriaceous, with satin sheen (as outer side); middle and hind tibia with numerous short spines; length of femur, tibia and basitarsus of middle leg 5.9, 9.4 and 11.4 times as long as their width, respectively; inner and outer middle tibial spurs 0.55 and 0.35 times as long as basitarsus; length of femur, tibia and basitarsus of hind leg 4.1, 7.6 and 9.1 times their width, respectively; inner and outer hind tibial spurs 0.55 and 0.40 times as long as basitarsus, respectively.

**Figure 59.**
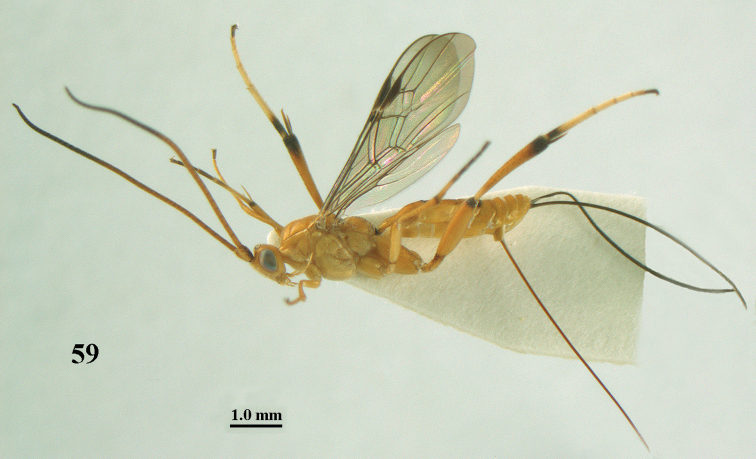
*Stantonia robustifemur* sp. n., ♀, holotype, habitus, lateral aspect.

*Metasoma*. First tergite gradually widened (Fig. 63), 2.3 times as long as its apical width, its surface finely granulate and rather dull apically slightly micro-sculptured; second and third tergites granulate, stout and rather dull; second suture straight and area behind nearly flat; length of ovipositor sheath 1.07 times as long as fore wing and 1.1 times as long as metasoma (Fig. 59).

**Figures 60–70.**
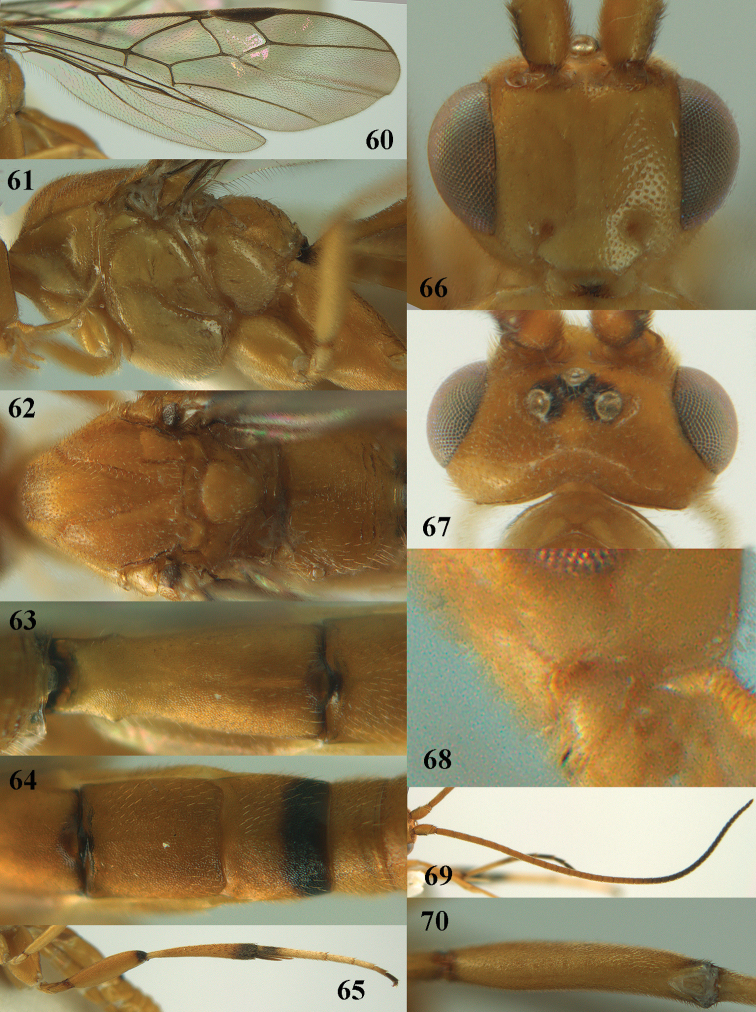
*Stantonia robustifemur* sp. n., ♀, holotype. **60** wings **61** mesosoma, lateral aspect **62** mesosoma, dorsal aspect **63** first metasomal tergite, dorsal aspect **64** second–fourth metasomal tergites, dorsal aspect **65** hind leg, lateral aspect **66** head, anterior aspect **67** head, dorsal aspect **68** detail of clypeus and malar space, lateral aspect **69** antenna **70** hind femur, ventral aspect.

*Colour*. Yellowish brown dorsally and remainder (including tegulum) pale brownish yellow; antenna brownish yellow, but outer side of scapus and pedicellus, and apical third of antenna dark brown; ovipositor sheath, base and apex of first tergite narrowly, base of second tergite slightly, apex of third tergite, telotarsi, hind basitarsus subbasally (but basally narrowly white), apex of hind femur, apex of middle tibia, apical 0.2 of hind tibia, and middle tarsus (but basitarsus largely yellowish), dark brown; basal ring of hind tibia and hind tarsus ivory, but basitarsus subbasally and telotarsus dark brown; apical fifth of fore wing slightly darkened and remainder subhyaline (Fig. 60); veins and pterostigma dark brown.

*Male*. Very similar to female: body length 5.0–5.5 mm, fore wing length 4.0–4.6 mm; antenna with 37(1), 38(2), 39(1) segments; length of hind femur 3.5 times its maximum width; length of first metasomal tergite 2.3–2.6 times its apical width.

#### Variation.

Female: body length 4.9–6.8 mm, fore wing length 4.2–5.2 mm; antenna with 40(1), 41(2) segments; length of hind femur 3.9–4.1 times its maximum width; inner spur of hind tibia 0.50–0.55 times as long as hind basitarsus; length of first metasomal tergite 1.9–2.6 times its apical width; medial length of second tergite 1.1–1.3 times its basal width and 1.3 times length of third tergite; length of ovipositor sheath 1.07–1.36 times as long as fore wing. The female from Yen Thuy is excluded from the type series because it has the second and third metasomal tergites more convex, resulting in a slenderer metasoma in dorsal view.

#### Distribution.

Vietnam (Ninh Binh (Cuc Phuong N.P).; Dak Lak, Chu Yang Sin N.P.; Dông Nai (Cát Tiên N.P.)).

### Stantonia ruficornis

Taxon classificationAnimaliaHymenopteraBraconidae

Enderlein, 1921

Fig. 71

Stantonia ruficornis Enderlein, 1921: 58; Shenefelt 1970: 267; van Achterberg 1987: 24; Long and Belokobylskij 2003: 396; Braet and Quicke 2004: 1561; Chen et al. 2004: 361–362, 532; Long and van Achterberg 2014: 408.

#### Material.

1 ♀ (RMNH), “N. Vietnam: Ninh Binh, Cuc Phuong N.P., n[ea]r entrance, c. 225 m, 1–15.v.2000, Mal. tr. II, Mai Phu Quy, RMNH’00”; 1 ♂ (RMNH), “C. Vietnam: Ha Tinh, Vu Quang N.P., 18°17'42"N, 105°25'34"E, 123 m, 5.iii.–15.iv.2011, Mal. trap 15, C. v. Achterberg & R. de Vries, RMNH’11”; 1 ♂ (IEBR), “Orgi.034”, “NW. Vietnam: Hoa Binh, Yen Thuy, forest, MT, 20°13'06"N, 105°34'11"E, 315 m, 10-20.iv.2002, K.D. Long”.

#### Diagnosis.

Antenna of ♀ 1.6–1.7 times as long as fore wing and its basal half yellowish brown, apically dark brown; vertex finely punctate or punctulate and interspaces distinctly wider than punctures; vertex and frons (especially of ♂) medially often dark brown; tegulum dark brown or infuscated; middle and lateral lobes of mesoscutum infuscate or dark brown medially; remainder of mesosoma brownish yellow; propodeum with coarse transverse rugae; fore wing infuscated apically; middle tarsus (except basitarsus) dark brown; apex of hind coxa more or less dark brown dorsally; apical half or quarter of hind femur dark brown; third hind tarsal segment ivory; outer side of hind femur rather shiny; ventrally hind femur rather matt and densely micro-sculptured ventrally; middle tarsus (except its basitarsus) more or less dark brown; first metasomal tergite darkened basally and approximately 3 times as long as its apical width; epipleuron of second tergite without a dark spot; length of ovipositor sheath 0.17–0.25 times as long as fore wing; length of fore wing 5.5–8.0 mm.

**Figure 71.**
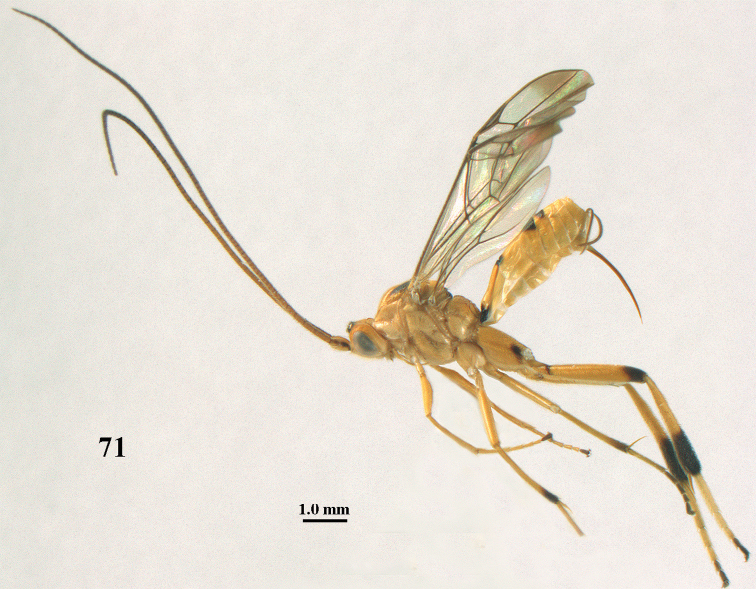
*Stantonia ruficornis* Enderlein, ♀, Vietnam, Cuc Phuong N.P., habitus, lateral aspect.

#### Distribution.

China (Jiangsu, Zhejiang, Hunan, Taiwan, Yunnan), West Malaysia, Philippines (Mindoro), Nepal (Braet and Quicke 2004) and Vietnam (Tonkin (Enderlein 1921, ♂); Hoa Binh, Lai Chau, Ha Tinh (Long and van Achterberg 2014); Ninh Binh).

### Stantonia sauteri

Taxon classificationAnimaliaHymenopteraBraconidae

Watanabe, 1932

Figs 72 , 73

Stantonia sauteri Watanabe, 1932: 188–189; Shenefelt 1970: 268; van Achterberg 1987: 24; Chen et al. 2004: 362, 532.

#### Type material.

Holotype, ♀ (SDEI), “Formosa [= Taiwan], Kankau (Koshun), vii.1012, H. Sauter”, “*Stantonia sauteri* Watanabe, Type”, “Holotypus”, “DEI-GYSHym 10631”. Specimen examined and photographed by Mr A.D. Liston.

#### Diagnosis.

Antenna of ♀ largely brownish yellow, without band of white or ivory segments submedially; vertex densely punctate and interspaces smaller than width of punctures and yellowish brown; mesosoma (including tegulum and humeral plate) brownish yellow, but middle lobe of mesoscutum with dark brown patch medially; fore wing infuscated apically; hind femur rugose ventrally, 5.2 times as long as wide and apically rather dark brown; hind tarsus (except telotarsus) ivory; length of first metasomal tergite approx. 2.5 times its apical width; base and apex of first tergite and base of second tergite yellowish brown; third and fourth tergites with dark brown patch; length of ovipositor sheath approx. 0.6 times as long as fore wing; length of fore wing approx. 5.5 mm. *Stantonia xiangqianensis* is similar, but has vertex sparsely punctate with interspaces much wider than punctures, hind femur smooth and shiny ventrally and first tergite approx. 3.7 times longer than wide posteriorly.

**Figures 72, 73.**
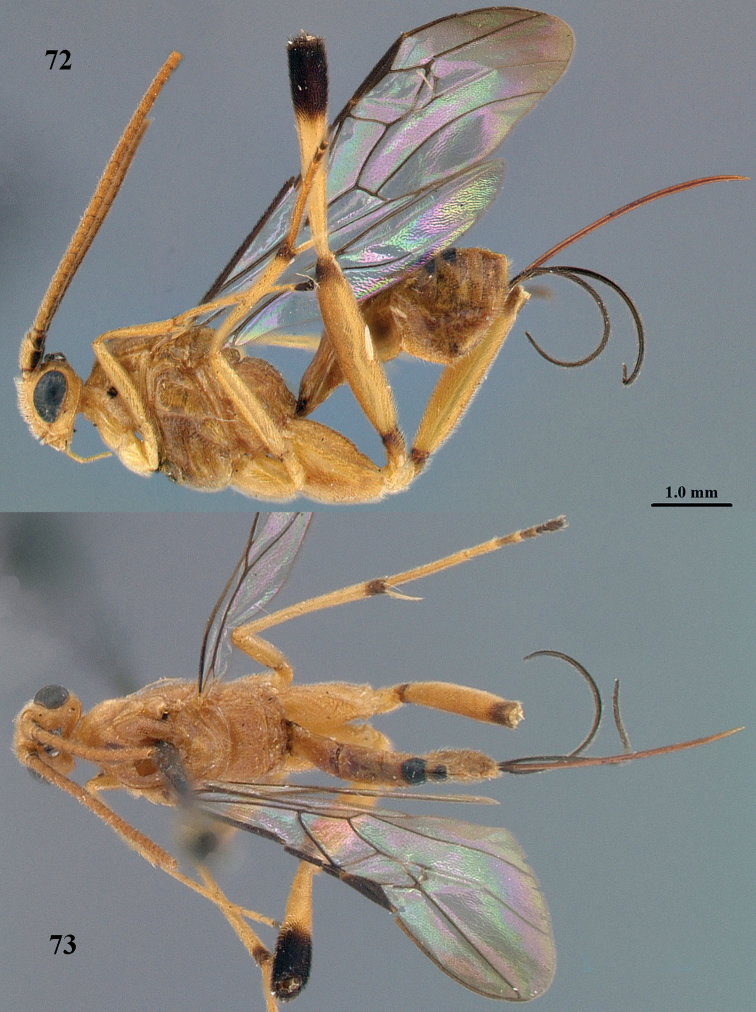
*Stantonia sauteri* Watanabe, ♀, holotype. **72** habitus, lateral aspect **73** habitus, dorsal aspect. Photos: A.D. Liston.

#### Distribution.

China (Taiwan).

### Stantonia spasskensis

Taxon classificationAnimaliaHymenopteraBraconidae

Belokobylskij, 1993

Figs 74 , 75–80

Stantonia spasskensis Belokobylskij, 1993: 97, 1998: 503.

#### Diagnosis.

**Figure 74.**
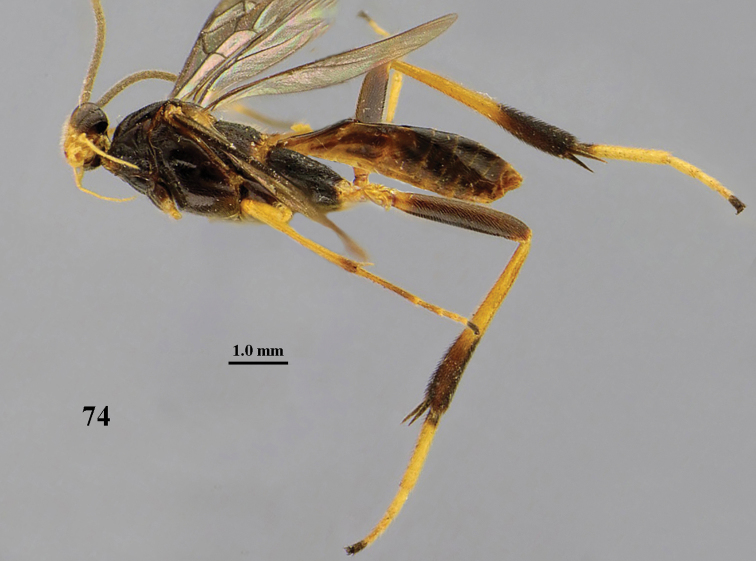
*Stantonia spasskensis* Belokobylskij, ♂, holotype, habitus, lateral aspect. Photo: K. Samartsev.

**Figures 75–80.**
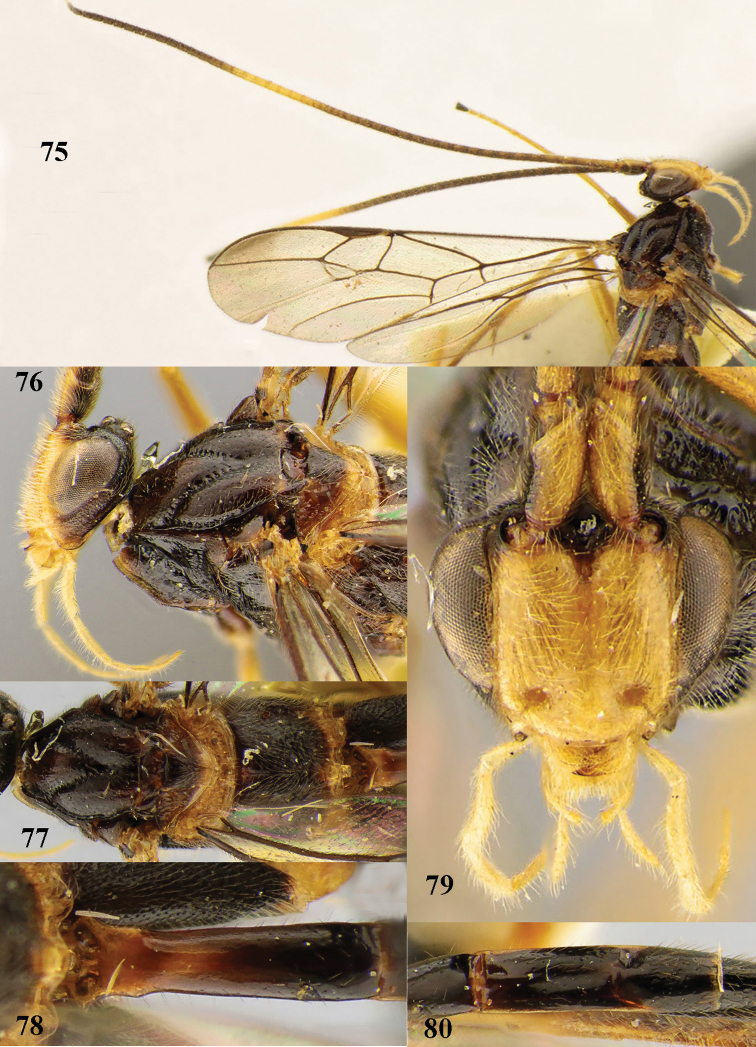
*Stantonia spasskensis* Belokobylskij, ♂, holotype. **75** antenna and wings **76** head, lateral aspect and mesosoma, latero-dorsal aspect **77** mesosoma, dorsal aspect **78** first metasomal tergite, dorsal aspect **79** head, anterior aspect **80** second and third metasomal tergites, dorsal aspect. Photos: K. Samartsev.

Antenna of ♂ with approximately 8 white or ivory segments (Fig. 75); anterior tentorial pits dorsally distinctly above lower level of eyes and malar space comparatively short (Fig. 79); temple coarsely rugose ventrally and (except for spaced punctures) largely smooth dorsally; mesosoma largely dark brown, only metanotum, propodeum and metapleuron posteriorly yellowish brown (Figs 74, 77); tegulum dark brown; anterior half of propodeum punctulate and largely smooth; hind femur dark brown medially; hind tarsus (except basally) whitish or ivory and moderately bristly setose; base of hind basitarsus dark brown; infuscation of apex of fore wing mainly restricted to marginal cell and just below it (Fig. 75); length of first tergite approx. 4 times its apical width (Fig. 78); second tergite dark brown and with shiny triangular area basally, its epipleuron largely rather fuzzy dark brown (Fig. 74); length of fore wing approx. 7.5 mm.

#### Distribution.

Far East Russia.

#### Notes.

The scanty material does not allow a conclusion about the validity of the taxon; the holotype may concern the melanistic male of *S. annulicornis* Enderlein, 1921. The male of *S. spasskensis* reported from Vietnam (Long and van Achterberg 2014) was re-examined and proved to belong to *S. annulicornis* Enderlein.

### Stantonia stilpnosoma

Taxon classificationAnimaliaHymenopteraBraconidae

Long & van Achterberg sp. n.

http://zoobank.org/1792CFA7-76D2-4759-84EB-284D98422434

Figs 81 , 82–92 , 93–94

#### Type material.

Holotype, ♀ (IEBR), “Orgi.004”, “N.W. Vietnam: Hoa Binh, Yen Thuy, orchard, MT 20°23'N, 105°36'E, 55 m, 1–10.ix.2001, K.D. Long”. Paratypes (2 ♀ + 4 ♂): 1 ♂ (IEBR), “Orgi.038”, “N.E. Vietnam, Phu Tho, Xuan Son N.P., 20.v.2005, P.Th. Nhi”; 1 ♀ (RMNH), “N. Vietnam: Ninh Binh, Cuc Phuong N.P., n[ea]r entrance, c. 225 m, 15.v.–27.v.2000, [Mal. tr.] I, Mai Phu Quy, RMNH’00”; 2 ♂ (RMNH, IEBR), id., but 1–15.v.2000, Malaise trap II; 1 ♀ + 1 ♂ (RMNH), id., 18.viii–17.ix.2000.

#### Diagnosis.

Antenna of ♀ 1.7–1.8 times as long as fore wing (of ♂ 2.1 times), without white or ivory segments and largely dark brown; anterior tentorial pits dorsally distinctly above lower level of eyes and malar space comparatively short (Fig. 88); frons brownish yellow medially; clypeus convex (Fig. 88); middle lobe of mesoscutum brownish yellow medially; mesopleuron ventrally and mesosternum yellowish brown; propodeum strongly shiny and with weak transverse rugae; vein r-m of fore wing partly pigmented (Fig. 82); fore wing hardly darkened apically (Fig. 82); hind femur slender, ventrally nearly entirely yellowish, finely punctate and interspaces smooth and shiny; hind coxa strongly shiny dorsally (Fig. 86); hind basitarsus moderately slender, whitish and usually erect bristly setose (Fig. 86), remainder dark brown; first metasomal tergite of ♀ strongly shiny and 3.3–3.6 times as long as its apical width; apex of first tergite and base of second tergite yellowish brown; second tergite of ♀ 1.6–1.7 times as long as wide; second metasomal suture straight and medial area behind suture flat or nearly so (Fig. 85); second epipleuron entirely yellow; third tergite distinctly punctate posteriorly; length of ovipositor sheath 0.10–0.17 times fore wing, distinctly less than half length of metasoma.

**Figure 81.**
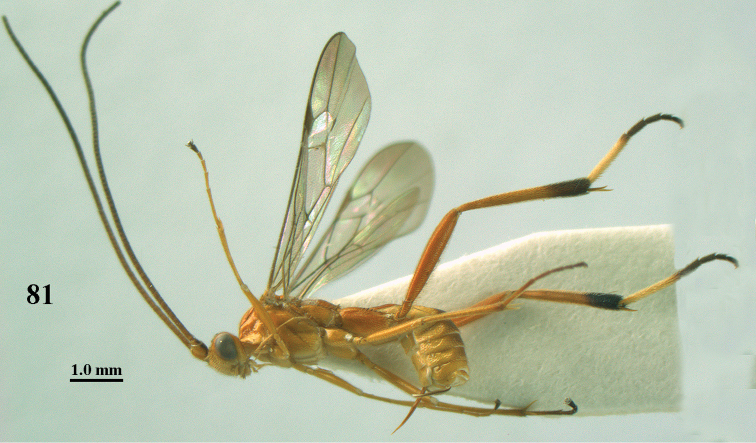
*Stantonia stilpnosoma* sp. n., ♀, paratype, Cuc Phuong N.P., habitus, lateral aspect.

**Figures 82–92.**
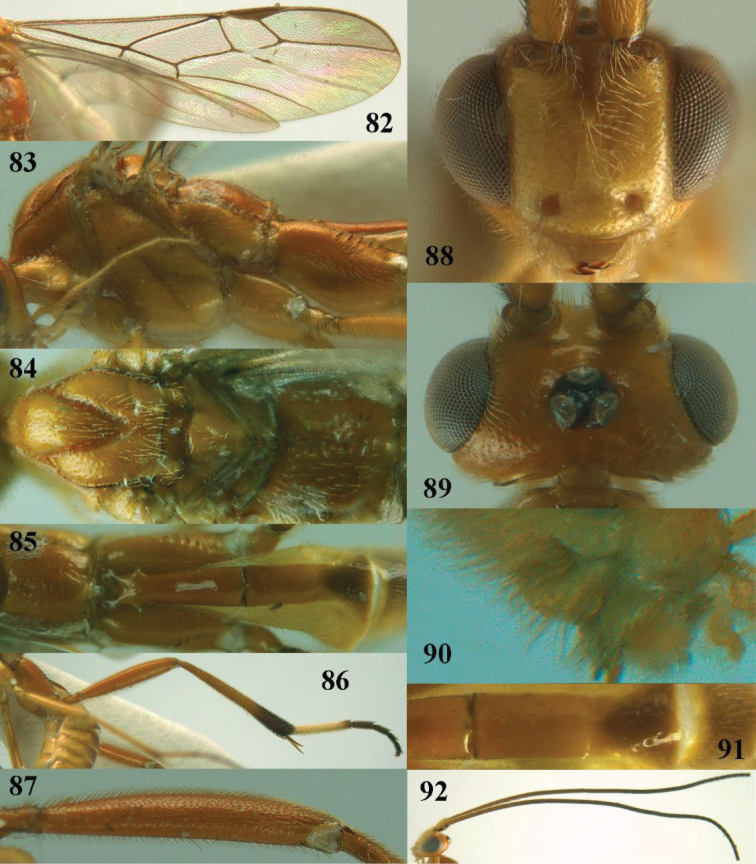
*Stantonia stilpnosoma* sp. n., ♀, paratype, Cuc Phuong N.P. **82** wings **83** mesosoma, lateral aspect **84** mesosoma, dorsal aspect **85** first–fourth metasomal tergites, dorsal aspect **86** hind leg, lateral aspect **87** hind femur, ventral aspect **88** head, anterior aspect **89** head, dorsal aspect **90** detail of clypeus and malar space, lateral aspect **91** second and third metasomal tergites, dorsal aspect **92** antenna.

**Figures 93–96.**
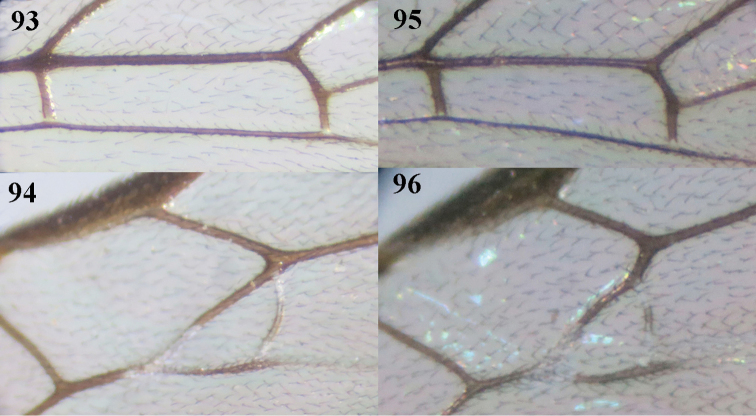
**93, 94** *Stantonia stilpnosoma* sp. n., ♀, holotype **95, 96**. *S. tianmushana* Chen, He & Ma, ♀, Vietnam, Vu Quang N.P. **93, 95** first subdiscal cell of fore wing **94, 96** second submarginal cell of fore wing.

The new species runs in the key by van Achterberg (1987) to *S. sumatrana* Enderlein, but differs by having the first metasomal tergite of ♀ 3.3–3.7 times as long as its apical width (2.4–2.7 times in *S. sumatrana*), ventrally hind femur smooth and shiny between punctures (coriaceous and dull), length of ovipositor sheath 0.10–0.15 times fore wing (0.16–0.19 times), and second and following hind tarsal segments dark brown (white or ivory, except dark telotarsus, rarely infuscated).

#### Description.

Holotype, female. Body length 5.4 mm, fore wing length 5.6 mm, ovipositor sheath 0.6 mm.

*Head*. Antenna with 46 segments but incomplete, at least 1.6 times as long as fore wing; middle segments twice as long as wide, third and fourth segments 3.3 and 2.3 times as long as wide, respectively, and third segment 1.2 times fourth segment; width of face 0.9 times height of face and clypeus combined (Fig. 88); maxillary palp 1.25 times as long as height of head; malar space 1.5 times as long as basal width of mandible; clypeus distinctly convex (Figs 88, 90); distance between tentorial pits twice as long as distance from pit to eye margin; in frontal view length of eye 2.4 times as long as its width; in dorsal view length of eye 2.5 times as long as temple; POL:OD:OOL = 2:3:7; distance between anterior and lateral ocellus 0.5 times OD (Fig. 88); face dull with sparse fine punctures and long setae; vertex and temple with sparse fine punctures; occipital flange wide and lamelliform.

*Mesosoma.* Length of mesosoma 1.4 times its height; pronotal side smooth dorsally and remainder sparsely finely punctate, medial sulcus with several crenulae anteriorly; notauli narrow and sparsely crenulate (Fig. 84); lobes of mesoscutum and scutellum with sparse fine punctures; precoxal sulcus narrow and finely crenulate (Fig. 83), area above precoxal sulcus almost smooth, area below precoxal sulcus with sparse fine punctures as metapleuron; propodeum shiny and largely smooth with basal medio-longitudinal carina and 2 transverse carinae medially (Fig. 84).

*Wings*. Fore wing: pterostigma 5.0 times as long as wide; r:2-SR:3-SR+SR1:r-m = 7:9:31:5; r issued behind middle of pterostigma; r-m present (Fig. 82); cu-a interstitial; basal half of CU1a mainly only pigmented; CU1b: 3-CU1 = 3:4. Hind wing: M+CU:1-M: 1r-m = 4:26:1; R1 with three distinct hamuli.

*Legs*. Ventrally hind femur punctate and interspaces smooth and shiny; length of femur, tibia and basitarsus of middle leg 10.2, 11.7 and 13.3 times as long as their width, respectively; inner and outer middle tibial spurs 0.5 and 0.4 times as long as basitarsus; length of femur, tibia and basitarsus of hind leg 4.9, 8.0 and 9.2 times their width, respectively; inner and outer hind tibial spurs 0.4 and 0.3 times as long as basitarsus, respectively.

*Metasoma*. First tergite almost parallel-sided, 3.3 times as long as its apical width, its surface largely smooth (Fig. 85); first tergite 1.4 times as long as propodeum; second tergite smooth (except for some punctures), elongate and shiny, 1.7 times longer than its basal width; length of ovipositor sheath 0.10 times as long as fore wing; ovipositor thick.

*Colour*. Yellowish brown; antenna brown but apically dark brown; tegulum and humeral plate pale yellow; stemmaticum, pterostigma, veins and middle tarsus dark brown or infuscated, but middle basitarsus yellowish basally; apical one fourth of hind tibia and second–fifth hind tarsal segments black; hind basitarsus ivory, but apically dark brown; fore wing slightly infuscated apically.

*Male.* Very similar to female: length of body 6.0–7.1 mm, of fore wing 3.3–6.0 mm; antenna with 55(1), 57(1) segments and 2.1 times as long as fore wing; length of femur, tibia and basitarsus of hind leg 4.8, 8.6 and 9.0 times their width, respectively; fore wing: r:2-SR:3-SR+SR1 = 8:10:27; CU1b: 3-CU1 = 3:7; hind wing: M+CU:1-M: 1r-m = 8:30:2; propodeum without or with basal medio-longitudinal carina and with 2–5 transverse carinae medially.

#### Variation.

Female: length of body 5.4–6.9 mm, of fore wing 5.3–6.0 mm; vein cu-a of fore wing antefurcal (Fig. 82) or interstitial; length of first tergite 3.3–3.6 times length of fore wing; length of ovipositor sheath 0.10–0.17 times fore wing; first tergite 3.3–3.6 times its apical width; length of mesosoma 1.4–1.5 times its height; length of first metasomal tergite 3.4–3.8 times its apical width; length of ovipositor sheath 0.10-0.17 times length of fore wing; fore wing slightly infuscated apically.

#### Distribution.

Vietnam (Phu Tho (Xuan Son N.P.); Hoa Binh; Ninh Binh (Cuc Phuong N.P.)).

#### Etymology.

Named after the very shiny (“stilpnos” is Greek for “glittering, glisterning”) body (“soma” in Greek) of this species.

### Stantonia sumatrana

Taxon classificationAnimaliaHymenopteraBraconidae

Enderlein, 1908

Figs 97 , 98

Stantonia sumatrana Enderlein, 1908: 110; Shenefelt 1970: 268; van Achterberg 1987: 24, 47–48; Braet and Quicke 2004: 1579; Chen et al. 2004: 362–363, 532.

#### Material.

2 ♀ (IZAS), China: Guangxi, Pingxiang, 230 m & Mt. Daqing, 600-700 m; 1 ♀ (ZISP), Vietnam: Tản Lĩnh, Ba Vi, 70 km NW Hanoi, 400 m, forest; 1 ♀ + 1 ♂ (NWUX), “SW. China: Yunnan, Yaoqu, Menglun, c. 540 m, 21°93'N, 101°26'E, 5.x.2010, Jiangli Tan, NWUX”; 1 ♂ (RMNH), “Vietnam: Ninh Thuân, Núi Chúa N.P., northeast part, Mal. traps, 90–150 m, 24–30.v.2007, C. v. Achterberg & R. de Vries, RMNH’07”; 1 ♂ (IEBR), id., but dry south part, 100–180 m, 22–29.v.2007.

#### Diagnosis.

**Figures 97, 98.**
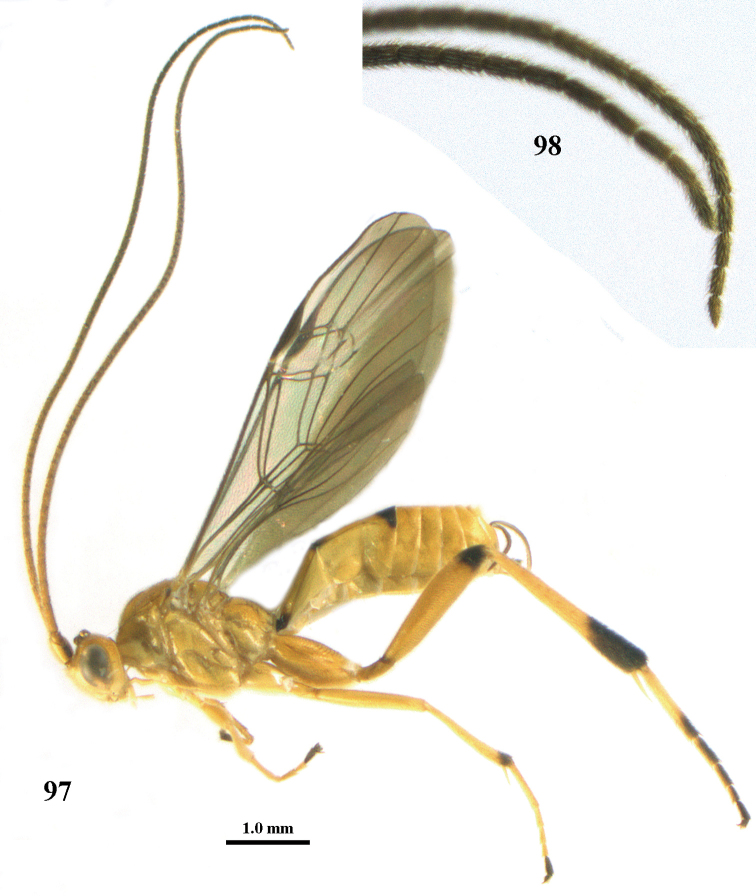
*Stantonia sumatrana* Enderlein, ♀, Malaysia, Sabah, Poring. **97** habitus, lateral aspect **98** apices of antennae.

Antenna of ♀ 1.7–1.8 times as long as fore wing, without white or ivory segments, largely dark brown and penultimate antennal segments of ♀ at least twice as long as wide (Fig. 98); anterior tentorial pits dorsally distinctly above lower level of eyes and malar space comparatively short; frons brownish yellow medially; clypeus convex; vertex finely to moderately punctate; middle lobe of mesoscutum brownish yellow medially; mesopleuron ventrally and mesosternum yellowish brown; propodeum with satin sheen and with coarse transverse rugae; vein r-m of fore wing partly pigmented (Fig. 97); fore wing hardly darkened apically (Fig. 97); humeral plate partly brown or dark brown; ventrally hind femur interspaces between punctures of ventral face micro-sculptured and rather matt, ventrally nearly entirely yellowish; hind femur slender (Fig. 97); hind coxa with satin sheen dorsally (Fig. 97); hind basitarsus moderately slender, apex brownish or dark brown and usually distinctly erect bristly setose (Fig. 97), remainder dark brown; first metasomal tergite of ♀ with satin sheen and 2.4–2.9 times as long as its apical width; apex of first tergite and base of second tergite yellowish brown; second tergite of ♀ 1.6–1.7 times as long as wide; second metasomal suture straight and medial area behind suture flat or nearly so; second epipleuron entirely yellow; length of ovipositor sheath 0.16–0.22 times fore wing, less than half length of metasoma; length of fore wing 4.7–6.0 mm.

#### Variation.

Apex of middle femur yellowish brown apically (also at inner side).

#### Distribution.

Indonesia (Sumatra, Java, Sulawesi), West Malaysia (van Achterberg 1987), Singapore, Australia (Northern Territory; Queensland), China (Hainan, Guangxi, Hunan, Yunnan), Philippines (Mindanao) (Braet and Quicke 2004), Vietnam (Ha Noi, Ba Vi, Tản Lĩnh; Ninh Thuan, Nui Chua). New record for Vietnam.

### Stantonia takeuchii

Taxon classificationAnimaliaHymenopteraBraconidae

(Watanabe, 1937)

Figs 99 , 100–110

Microtypus takeuchii Watanabe, 1937: 95.
Stantonia takeuchii ; Shenefelt, 1970: 268; Watanabe 1957: 45; Belokobylskij 1998: 503.

#### Type material.

Holotype, ♀ (ECHU), “[Japan: Honshu], Kyoto, 21.ix.1925, Takeuchi” (with extra label in Japanese), “*Microtypus takeuchii* Watanabe, Type”.

**Figure 99.**
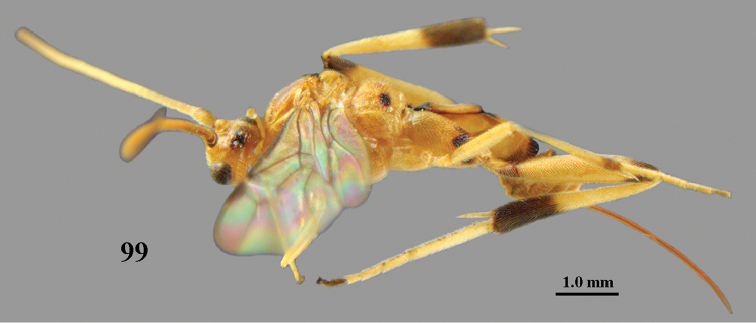
*Stantonia takeuchii* (Watanabe), ♀, holotype, habitus, lateral aspect.

#### Additional material.

1 ♀ (RMNH), “China: Zhejiang, Hangzhou, 7.vii.1985, no. 851849, He Junhua”.

#### Diagnosis.

**Figures 100–110.**
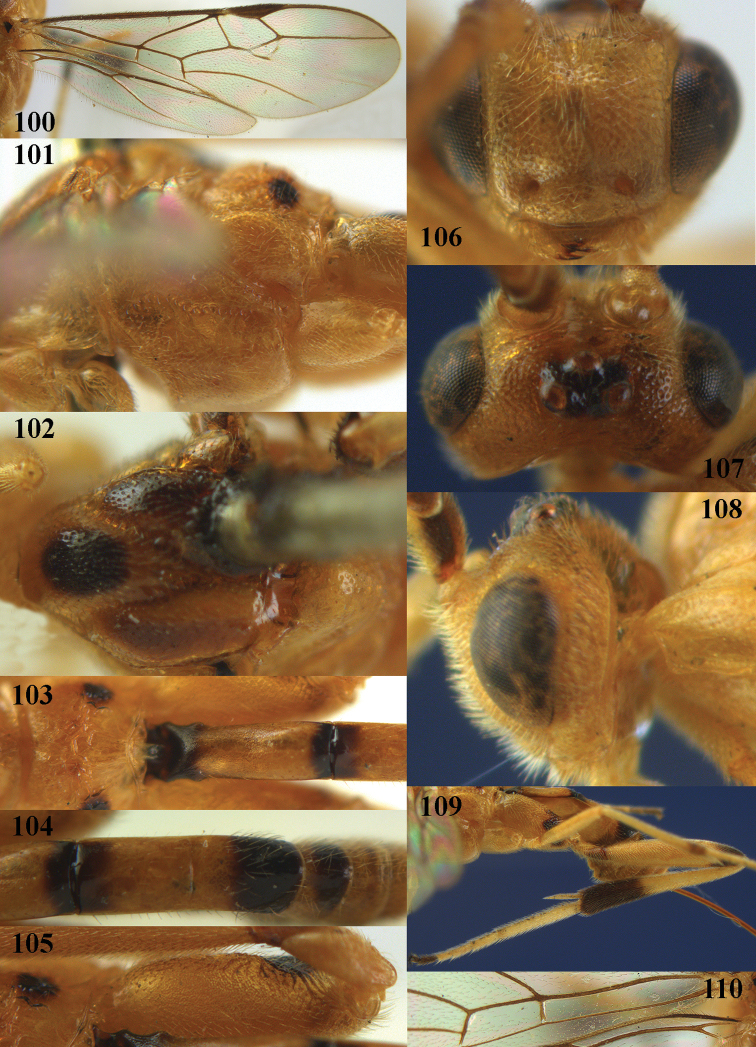
*Stantonia takeuchii* (Watanabe), ♀, holotype. **100** wings **101** mesosoma, lateral aspect **102** mesosoma, dorsal aspect **103** propodeum and first metasomal tergite, dorsal aspect **104** second and third metasomal tergites, dorsal aspect **105** hind coxa, dorsal aspect **106** head, anterior aspect **107** head, dorsal aspect **108** head, lateral aspect **109** hind leg, lateral aspect **110** detail of submedial and first subdiscal cells of fore wing.

Antenna of ♀ yellowish submedially; vertex strongly punctate and interspaces approximately as wide as punctures or less (Fig. 107); anterior tentorial pits dorsally distinctly above lower level of eyes and malar space comparatively short (Fig. 106); face strongly convex; clypeus convex (Fig. 106); middle lobe of mesoscutum largely dark brown; lateral lobes of mesoscutum more or less infuscate medially; mesopleuron ventrally and mesosternum yellowish brown; anterior half of propodeum rugose and posterior half smooth; inner half of humeral plate dark brown; vein r-m of fore wing partly pigmented (Fig. 100); fore wing narrowly darkened apically (Fig. 100); hind femur slender, densely finely sculptured and rather matt ventrally (Fig. 109); hind coxa with dark brown patch latero-apically; length of first tergite approx. 3.3 times its apical width and tergite hardly narrowed behind spiracles (Fig. 103), its surface largely superficially granulate; base and apex of first tergite and base of second tergite dark brown; second metasomal suture straight and medial area behind suture flat or nearly so (Fig. 104); length of ovipositor sheath 0.5–0.6 times as long as fore wing; length of body approx. 6 mm.

#### Description.

Holotype, ♀. Body length 6.1 mm, fore wing length 5.8 mm, ovipositor sheath missing, but length of ovipositor in normal position 3.2 mm.

*Head*. Antenna broken; third and fourth antennal segments 3.4 and 2.5 times as long as wide, respectively, and third segment 1.4 times as long as fourth segment; width of face equal to height of face and clypeus combined (Fig. 106); maxillary palp 1.2 times as long as height of head; clypeus distinctly convex and punctate (Fig. 106); malar space 1.1 times as long as mandible width; distance between large tentorial pits 1.9 times as long as distance between pit and eye margin; in anterior view length of eye 2.5 times as long as wide; in dorsal view length of eye 2.2 times as long as temple; POL:OD:OOL = 11:9:16; distance between anterior and lateral ocellus 0.8 times OD (Fig. 107); face convex, rather densely and coarsely punctate, smooth interspaces about equal to diameter of punctures and with medium-sized setae; frons laterally and vertex densely punctate (interspaces somewhat narrower than punctures), interspaces smooth and area directly behind stemmaticum depressed; frons medially smooth; stemmaticum strongly protruding; temple with satin sheen and with mainly rugose-coriaceous; occipital flange wide lamelliform.

*Mesosoma.* Length of mesosoma 1.2 times as long as high; pronotal side largely smooth except some spaced coarse punctures and coarsely and widely crenulate medial sulcus, subposteriorly with two crenulate branches and posteriorly narrowly crenulate; precoxal sulcus narrow and finely crenulate, complete and with wide flange posteriorly (Fig. 101), mesopleuron remotely coarsely punctate, smooth interspaces much wider than punctures; metapleuron coarsely punctate, with smooth interspaces approximately as wide as punctures; notauli rather narrow and moderately crenulate; mesoscutum and scutellum rather coarsely punctate, with smooth interspaces equal to width of punctures (middle lobe) or wider (lateral lobes and scutellum; Fig. 102); propodeum rather shiny, anterior half coarsely reticulate-rugose and posteriorly half mainly smooth.

*Wings*. Fore wing (Fig. 100): pterostigma 3.9 times as long as wide; second submarginal cell petiolate; r:2-SR:3-SR+SR1:r-m = 20:25:86:16; r issued submedially from pterostigma; r-m submedially distinctly sclerotized; cu-a slightly antefurcal (Fig. 100); basal 0.7 of CU1a more or less sclerotized; CU1b: 3-CU1 = 10:13. Hind wing: M+CU:1-M: 1r-m = 19:59:5.

*Legs*. Anterior half of hind coxa punctate-rugulose and posterior half coarsely rugose dorsally (Fig. 105); ventrally hind femur rather matt, largely rugulose-coriaceous; length of femur, tibia and basitarsus of middle leg 6.3, 10.7 and 12.2 times as long as their width, respectively; inner and outer middle tibial spurs 0.40 and 0.35 times as long as basitarsus; length of femur, tibia and basitarsus of hind leg 5.3, 7.8 and 7.6 times their width, respectively; hind basitarsus rather adpressed setose; inner and outer hind tibial spurs 0.50 and 0.35 times as long as basitarsus, respectively.

*Metasoma*. First tergite hardly narrowed behind spiracles (Fig. 103), 3.3 times as long as its apical width, its surface superficially finely granulate and with satin sheen; second tergite convex and smooth anteriorly, remainder superficially granulate, rather elongate, 1.5 times longer than its basal width and with satin sheen; second suture straight and medial area behind it nearly flat; ovipositor sheath missing, considering position and length of ovipositor 0.56 times as long as fore wing and approximately as long as metasoma (Fig. 99).

*Colour*. Yellowish brown; inner half of humeral plate dark brown, remainder of plate and tegulum yellowish brown; tibial spurs, fore and middle legs (but telotarsi, third and fourth middle tarsal segments and apex of hind tibia dark brown) pale yellowish; remainder of hind tarsus ivory (Fig. 99); basal half of antenna (but scapus and pedicellus dark brown laterally) brownish yellow; lateral lobes of mesoscutum slightly darkened medially; stemmaticum, middle lobe of mesoscutum, pair of lateral patches on propodeum, base and apex of first metasomal tergite, base of second tergite, apex of third and fourth segments, patch on hind coxa subapically, hind trochantellus, apex of hind femur, apical third of hind tibia, large patch on second epipleuron and apical half of third epipleuron dark brown; apex of fore wing anteriorly slightly darkened and remainder subhyaline (Fig. 100); veins and pterostigma dark brown.

#### Distribution.

Japan (Honshu, Kyushu), China (Taiwan (Belokobylskij 1998), *Zhejiang).

#### Notes.

Very similar to *S. sauteri* Watanabe and differs mainly by the partly dark brown basal metasomal tergites and hind coxa. The pair of dark brown patches of the propodeum is absent in the specimen from Hangzhou.

### Stantonia tianmushana

Taxon classificationAnimaliaHymenopteraBraconidae

Chen, He & Ma, 2004

Figs 95 , 96 , 111

Stantonia sp. C Braet & Quicke, 2004: 1522.
Stantonia tianmushana Chen, He & Ma, 2004: 364–365, 533.

#### Material.

1 ♀ (RMNH), N. Vietnam: Vinh Phu, Tam Dao, 700 m, *Pinus* forest, 14.ii.1990, S.A. Belokobylskij; 1 ♀ (ZISP), id., but 1000 m; 1 ♀ (ZISP), N. Vietnam, Ba Vi, 70 km NW Ha Noi, 400 m; 1 ♀ (RMNH), “N. Vietnam: Hai Phong, Cat Ba N.P., 95 m, 20°48'2"N, 107°0'18"E, 18–24.x.2009, Mal. tr., C. v. Achterberg & R. de Vries, RMNH’09”; 1 ♀ (IEBR), “Orgi.070”, “NC Vietnam: Ha Tinh, Vu Quang N.P., 4.x.2009, K.D. Long”.

#### Diagnosis.

**Figure 111.**
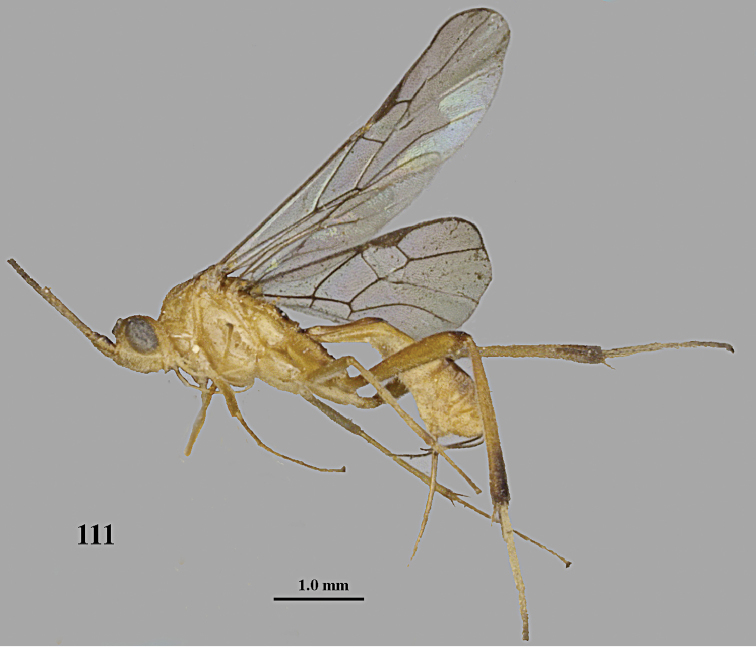
*Stantonia tianmushana* Chen, He & Ma, ♀, holotype, habitus, lateral aspect. Photo: Jiachen Zhu.

Antenna of ♀ without white or ivory segments and largely dark brown; anterior tentorial pits dorsally above lower level of eyes and malar space comparatively short; frons brownish yellow medially; clypeus convex; vertex finely to moderately punctate; middle lobe of mesoscutum brownish yellow medially; mesopleuron ventrally and mesosternum yellowish brown; propodeum with satin sheen and with coarse transverse rugae; vein r-m of fore wing partly pigmented (Fig. 111); fore wing subhyaline apically (Fig. 111); humeral plate partly brown or dark brown; ventrally hind femur densely micro-sculptured and matt, ventrally nearly entirely yellowish; hind femur slender (Fig. 111); hind coxa with satin sheen dorsally; hind basitarsus slender and rather adpressed setose (Fig. 111); first metasomal tergite of ♀ 3.0–3.6 times as long as its apical width; apex of first tergite and base of second tergite yellowish brown; second tergite of ♀ 1.6–1.7 times as long as wide; second metasomal suture straight and medial area behind suture flat or nearly so; second epipleuron entirely yellowish; length of ovipositor sheath 0.25–0.32 times fore wing, approximately half as long as metasoma; length of body 4–5 mm.

#### Variation.

First tergite 3.0-3.6 times as long as wide apically.

#### Distribution.

China (Zhejiang), *Vietnam (Vinh Phuc (Tam Dao N.P.); Ha Noi (Ba Vi N.P.); Ha Tinh (Vu Quang N.P.)). New record for Vietnam.

### Stantonia vietnamica

Taxon classificationAnimaliaHymenopteraBraconidae

van Achterberg sp. n.

http://zoobank.org/D6DAA97B-F5B1-4A9E-BDE0-BE4A13FB1EF6

Figs 112 , 113–122

Stantonia spasskensis ; Braet & Quicke, 2004: 1578–1579.

#### Type material.

Holotype, ♀ (RMNH), “S. Vietnam: Dak Lak, Chu Yang Sin N.P., n[ea]r dam, 740–940 m, 1–10.vi.2007, Mal. traps, C. v. Achterberg & R. de Vries, RMNH’07”. Paratypes (3 ♀): 1 ♀ (IEBR), same data as holotype; 1 ♀ (RMNH), “C. Vietnam: Ha Tinh, Vu Quang N.P., 18°19'47"N, 105°26'28"E, 66 m, 4.iii.–15.iv.2011, Mal. trap 9, C. v. Achterberg & R. de Vries, RMNH’11”; 1 ♀ (ZISP), “Vietnam, pr[ovince] Ha Son Binh, Da Bac, forest bamboo, 22.x.1990, Tuly & Belokobylskij”.

#### Diagnosis.

**Figure 112.**
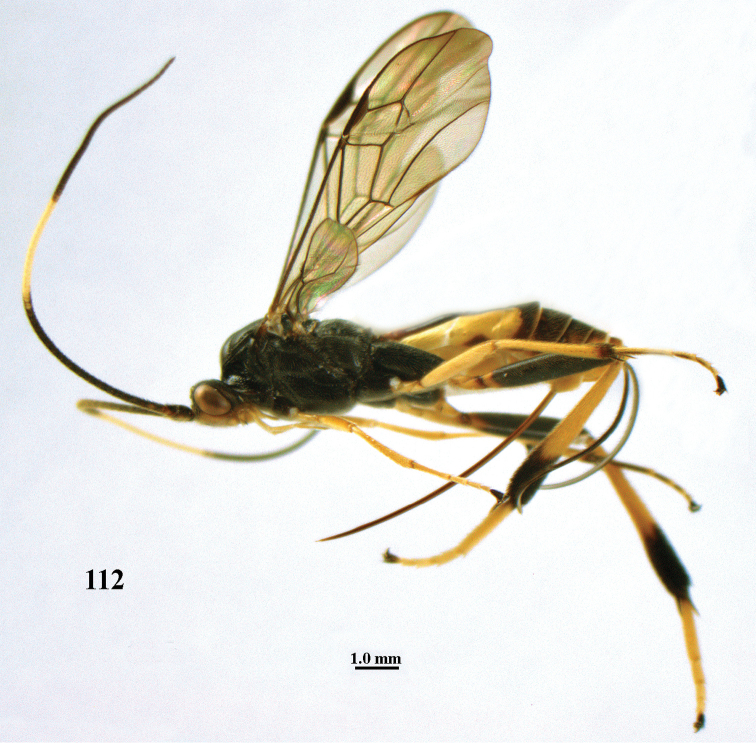
*Stantonia vietnamica* sp. n., ♀, holotype, habitus, lateral aspect.

**Figures 113–122.**
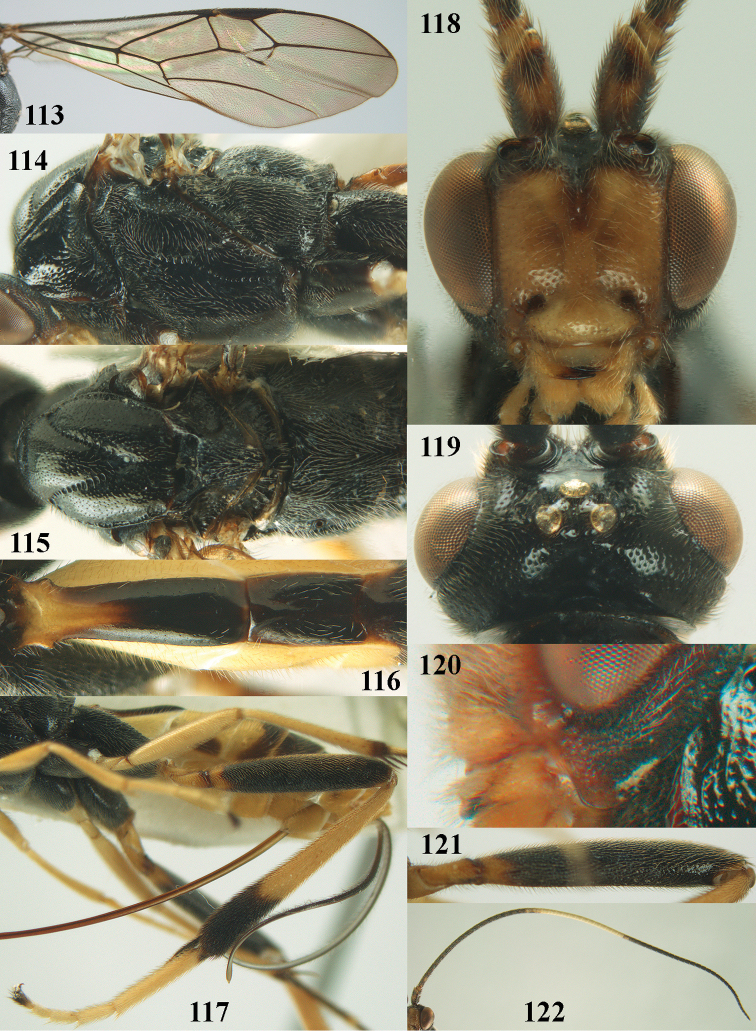
*Stantonia vietnamica* sp. n., ♀, holotype. **113** wings **114** mesosoma, lateral aspect **115** mesosoma, dorsal aspect **116** first–second metasomal tergites, dorsal aspect **117** hind leg, lateral aspect **118** head, anterior aspect **119** head, dorsal aspect **120** detail of clypeus and malar space, lateral aspect **121** hind femur, ventral aspect **122** antenna.

Antenna with a submedial band consisting of 14–17 ivory or white segments contrasting with blackish or dark brown basal third of antenna (Fig. 112); clypeus moderately convex (Fig. 118); mesosoma entirely black; tegulum and humeral plate dark brown; fore wing evenly infuscated (Fig. 113); hind tarsus (except narrowly basally) whitish or ivory; middle tarsus conspicuously bristly setose; hind femur (except basally) and more or less middle coxa black or dark brown dorsally; first metasomal tergite 3.4–3.6 times as long as wide apically; epipleuron of second metasomal tergite with an isolated and well defined dark brown spot (Fig. 117); second metasomal tergite dark brown to brownish yellow antero-dorsally; length of ovipositor sheath 0.5–0.6 times as long as fore wing; length of fore wing 7.9–9.2 mm.

The new species runs in the key by van Achterberg (1987) to *S. annulicornis* Enderlein, but differs by having the face yellowish brown and rather densely punctate (pale yellowish and sparsely finely punctate in *S. annulicornis*), clypeus partly convex (rather flat), scapus partly infuscated (pale yellow), antenna with 14–17 white or ivory segments submedially (11–12 segments), fore wing evenly slightly infuscate (only apically distinctly infuscate), tegulum dark brown (pale brown or yellowish), and propodeum black medially (more or less yellowish brown or brown).

#### Description.

Holotype, ♀. Body length 9.7 mm, fore wing length 9.2 mm, ovipositor sheath 4.9 mm.

*Head*. Antenna with 61 segments and 1.6 times as long as fore wing; middle antennal segments with distinct false division medially and 1.8 times as long as wide; third, fourth and penultimate antennal segments 4.5, 2.9 and 1.8 times as long as wide, respectively, and third segment 1.6 times as long as fourth segment; width of face 0.9 times height of face and clypeus combined (Fig. 118); maxillary palp 1.5 times as long as height of head; malar space 0.7 times as long as mandible width, largely coriaceous and with distinct groove; distance between tentorial pits 2.2 times as long as distance between pit and eye margin; in anterior view length of eye 2.2 times as long as wide; in dorsal view length of eye 3.5 times as long as temple; POL:OD:OOL = 8:10:15; distance between anterior and lateral ocellus 0.5 times OD (Fig. 119); face remotely and moderately punctate, interspaces smooth and much wider than punctures and medium-sized setae; vertex finely remotely punctate and directly behind stemmaticum steeply depressed; temple matt, granulate, punctate and postero-ventrally with rugae; occipital flange wide and lamelliform.

*Mesosoma.* Length of mesosoma 1.5 times as long as high; pronotal side with complete Y-shaped crenulate grooves, postero-ventrally connected to crenulate border, largely smooth dorsally, partly coarsely punctate and with some rugae ventrally and remainder sparsely finely punctate (Fig. 114); notauli rather narrow and moderately crenulate, but widened posteriorly and ending far in front of scutellar sulcus (Fig. 115); scutellar sulcus smooth, except for some remnants of crenulae posteriorly; mesoscutum and scutellum remotely finely punctate (Fig. 115); precoxal sulcus complete and rather narrowly crenulate (Fig. 114), mesopleuron sparsely finely punctate, but mainly smooth near precoxal sulcus; metapleuron rather densely punctate; propodeum spaced punctate with interspaces smooth and at least as wide as diameter of punctures, rather dull and with few weak transverse rugae (Fig. 115).

*Wings*. Fore wing (Fig. 113): first discal cell truncate anteriorly; pterostigma 5.0 times as long as wide; r:2-SR:3-SR+SR1:r-m = 20:19:67:10; second submarginal cell narrowly petiolate; r issued behind middle of pterostigma; r-m largely sclerotized; cu-a interstitial; basal three-quarters of CU1a sclerotized; CU1b: 3-CU1 = 10:23, CU1b oblique, distinctly diverging posteriorly from cu-a. Hind wing: M+CU:1-M: 1r-m = 6:21:2; R1 with three distinct hamuli; area in front of cu-a and behind it glabrous.

*Legs*. Ventrally hind femur rugose, but posteriorly becoming obsolescent, with satin sheen (as outer side); length of femur, tibia and basitarsus of middle leg 5.5, 9.3 and 10.0 times as long as their width, respectively; inner and outer middle tibial spurs 0.45 and 0.30 times as long as basitarsus; middle tarsus very bristly; length of femur, tibia and basitarsus of hind leg 5.0, 8.3 and 6.4 times their width, respectively; inner and outer hind tibial spurs 0.50 and 0.35 times as long as basitarsus, respectively.

*Metasoma*. First tergite parallel-sided, 3.6 times as long as its apical width, its surface with satin sheen, largely smooth (except some superficial micro-sculpture and some punctures; Fig. 116); second and third tergites smooth (except some punctures and superficial micro-sculpture), and rather dull, except a shiny triangular basal area; second tergite 1.7 times longer than its basal width; length of ovipositor sheath 0.51 times as long as fore wing and 0.9 times as long as metasoma (Fig. 112).

*Colour*. Black; antenna dark brown but 3 basal antennal segments with pale brownish or greyish spots, 33^rd^ segment pale brown, 17^th^–18^th^ segments partly ivory and 19^th^–32^nd^ segments white; tegulum, humeral plate, apex of middle tibia, patch on second epipleuron, third tergite (except antero-laterally), fourth and fifth tergites largely, sixth tergite dorsally and ovipositor sheath dark brown; middle tibial spurs, inner apex of middle femur, seventh and eight tergites dorsally, brown; tarsi ivory, but telotarsi black, hind basitarsus basally narrowly blackish and middle tarsus dark brown ventrally; coxae, hind femur (except basally), apical third of hind tibia, hind tibial spurs, first (except basal triangle) and second tergites dorsally, black; remainder of legs and of metasoma, palpi and clypeus pale yellowish; face brownish yellow; entire fore wing infuscated; veins and pterostigma dark brown.

*Male*. Unknown.

#### Variation.

Length of body 7.2–9.7 mm; length of fore wing 7.0–9.2 mm; antenna with 55(1), 61(2) segments; length of first metasomal tergite 3.6–3.7 times its apical width; length of ovipositor sheath 0.51–0.54 times fore wing; 18^th^–30^th^ or –32^nd^, or 19^th^–32^nd^ antennal segments white.

#### Distribution.

Vietnam (Hoa Binh; Ha Tinh (Vu Quang N.P.); Dak Lak (Chu Yang Sin N.P.)).

#### Notes.

A male from Thanh Son (RMNH) has 14 pale antennal segments, yellow mesopleuron, mesosternum, middle coxa and middle of propodeum, and probably belongs to a related species. Except for having less pale antennal segments than the examined holotype of *S. spasskensis*, it differs by having vein cu-a of fore wing distinctly postfurcal (slightly antefurcal in *S. vietnamica*), less extensive infuscation of fore wing (most of apex of fore wing), and base of T1 yellowish-brown (ivory).

### Stantonia xiangqianensis

Taxon classificationAnimaliaHymenopteraBraconidae

Chen, He & Ma, 2004

Fig. 123

Stantonia sp. B Braet & Quicke, 2004: 1522.
Stantonia xiangqianensis Chen, He & Ma, 2004: 365–367, 532; Long and van Achterberg 2014: 408.

#### Material.

1 ♂ (RMNH), “S. China: Hunan, n[ea]r Zhangjiajie, Badagong Mts, Bamaoxi, 2–3.vi.2009, 540 m, X.-Y. Li, RMNH’09”.

#### Diagnosis.

**Figure 123.**
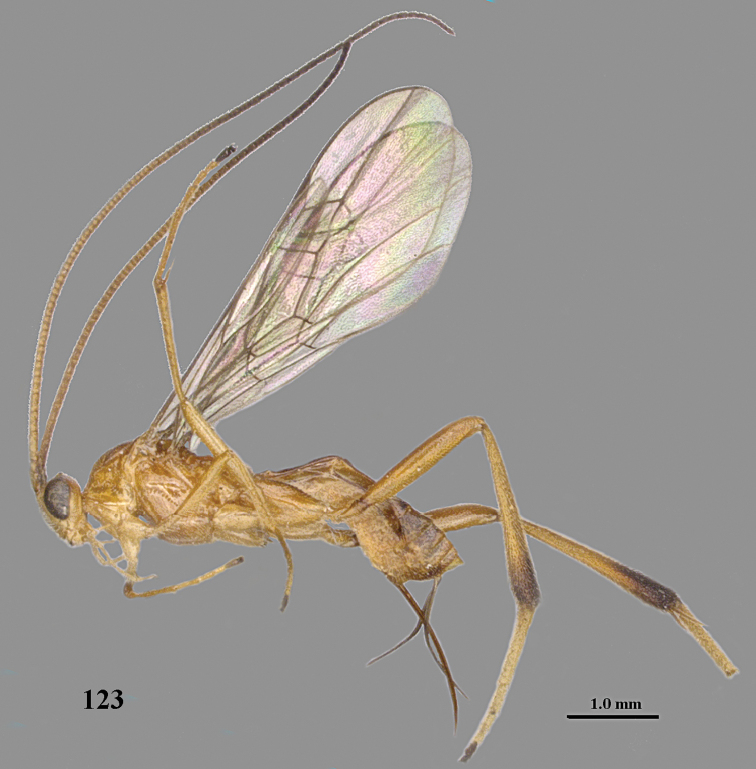
*Stantonia xiangqianensis* Chen, He & Ma, ♀, holotype, habitus, lateral aspect. Photo: Jiachen Zhu.

Basal half of antenna yellowish and apical half darkened, antenna 1.6 times as long as fore wing; vertex finely spaced punctate and interspaces distinctly wider than punctures and yellowish brown; mesosoma entirely yellowish brown; inner half of humeral plate dark brown, remainder of plate and tegulum yellowish brown; propodeum medio-anteriorly sparsely punctate anteriorly; fore wing moderately infuscated apically; vein 3-SR+SR1 approx. 3.7 times as long as vein r; hind femur partly smooth and shiny ventrally, slender and apically yellowish brown; hind tarsus (except telotarsus) ivory or white; length of first metasomal tergite approx. 3.7 times its apical width; epipleuron of second tergite partly darkened (Fig. 123); apices of first and third metasomal tergites brownish yellow; length of ovipositor sheath 0.5–0.6 times as long as fore wing and somewhat longer than metasoma; length of fore wing approximately 7 mm.

#### Distribution.

China (Hunan, Guizhou).

#### Notes.

Very similar to *S. issikii* Watanabe, 1932, and differs mainly by the colour and shape of the tergites and the longer vein 3-SR+SR1 of fore wing.

## Supplementary Material

XML Treatment for Stantonia

XML Treatment for Stantonia angustata

XML Treatment for Stantonia annulicornis

XML Treatment for Stantonia brevicaudata

XML Treatment for Stantonia chaoi

XML Treatment for Stantonia clappae

XML Treatment for Stantonia dickyyui

XML Treatment for Stantonia gracilis

XML Treatment for Stantonia granulata

XML Treatment for Stantonia issikii

XML Treatment for Stantonia qui

XML Treatment for Stantonia robustifemur

XML Treatment for Stantonia ruficornis

XML Treatment for Stantonia sauteri

XML Treatment for Stantonia spasskensis

XML Treatment for Stantonia stilpnosoma

XML Treatment for Stantonia sumatrana

XML Treatment for Stantonia takeuchii

XML Treatment for Stantonia tianmushana

XML Treatment for Stantonia vietnamica

XML Treatment for Stantonia xiangqianensis
